# Gαi1/3 Is a Novel Regulatory Target for RANKL Signal Transduction and Osteoporosis

**DOI:** 10.1002/advs.202510836

**Published:** 2026-02-12

**Authors:** Chaowen Bai, Mingchao Zhang, Le Liu, Lide Tao, Chenyang Xu, Hao Chai, Xin Shi, Yin Wang, Xiaopei Zhang, Qiong Chen, Dong Liu, Jinyu Bai, Chang She, Xiaozhong Zhou, Cong Cao, Huajian Shan

**Affiliations:** ^1^ Department of Orthopedics The Second Affiliated Hospital of Soochow University Suzhou China; ^2^ Department of Endocrinology The Second Affiliated Hospital of Soochow University Suzhou China; ^3^ Institution of Neuroscience Soochow University Suzhou China; ^4^ Shanxi Key Laboratory of Rheumatism Immune Microecology The Second Affiliated Hospital of Shanxi Medical University Taiyuan China

**Keywords:** Gαi1/3, osteoporosis, RANKL, signal transduction

## Abstract

Osteoporosis, characterized by progressive bone loss and increased fracture risk, is a growing concern as the population ages. Current treatments, though advanced, remain limited, underscoring the necessity for novel therapeutic targets. Recent studies have shown that the immune system plays a key role in osteoporosis, with osteoclasts driving bone resorption. Gαi proteins, critical mediators of immune signaling, are implicated in osteoclastogenesis, but their precise role remains unclear. It is demonstrated that Gαi1/3 was highly expressed in osteoclast precursors and mature osteoclasts, with elevated levels in the bone marrow of osteoporotic patients and ovariectomized (OVX) mice. Conditional knockout of Gαi1/3 in osteoclast precursors mitigates OVX‐induced bone loss, improving bone mass and structure. Gαi1/3‐deficient bone marrow monocytes and macrophages exhibit impaired osteoclast formation and reduced bone resorption. In contrast, overexpression of Gαi1/3 enhances osteoclast differentiation and function. In addition, the 173 Asp residue of Gαi3 is identified as crucial for RANK‐TRAF6 binding during RANKL signaling. Inhibition of Gαi1/3 mimics the protective effects of denosumab, a treatment for osteoporosis, in mice. The findings position Gαi1/3 as a critical regulator of osteoclastogenesis and suggest it as a promising therapeutic target for osteoporosis.

## 1 Introduction

Osteoporosis is a systemic skeletal disorder that affects bone tissue, characterized by decreased bone mass, disruption of bone microstructure, and increased bone fragility, which elevates the risk of fractures [1, 2, 3]. As the elderly population continues to grow, the incidence of osteoporosis has risen in recent years, with the prevalence exceeding 50% in women over 65 years of age. Despite advancements in osteoporosis treatments, including denosumab and romosozumab, these therapies exhibit certain limitations, including significant side effects [4]. Therefore, further elucidation of the pathophysiology of osteoporosis and the exploration of new therapeutic targets are essential, as they are crucial for improving treatments efficacy and enhancing patients quality of life [1].

Recent studies have established that bone tissue has been recognized as an “osteoimmune” organ, serving as a locomotor organ, a mineral reservoir, and a primary lymphoid organ responsible for maintaining hematopoietic stem cells [5]. Bone and mineral homeostasis relies on the coordinated activity of osteoclasts, osteoblasts, and osteocytes, yet pathological conditions can disrupt this balance through aberrant or prolonged immune responses [5]. In osteoporosis, monocytes differentiate into osteoclasts in response to increased demand, contributing to bone loss. In postmenopausal osteoporosis, estrogen deficiency not only directly affects bone tissue but also alters immune cell activity, further exacerbating bone resorption [6]. Given that osteoclasts belong to the monocyte/macrophage lineage, they interact closely with B and T lymphocytes, and their differentiation is regulated by receptor activator of nuclear factor κB ligand (RANKL), which is also produced by lymphocytes and regulates lymphopoiesis [5, 7, 8]. These findings underscore the critical role of the immune system in osteoporosis pathogenesis.

Gαi proteins (G protein alpha i subunits) are essential mediators of inhibitory signaling within the G protein‐coupled receptor (GPCR) system [9, 10]. The three primary Gαi subunits, Gαi1, Gαi2, and Gαi3, share 85%–95% amino acid sequence identity and exhibit overlapping expression patterns. These subunits are widely expressed in the immune system, skeletal tissue, and endothelium [11, 12, 13]. Notably, Gαi1/3 interact with CD14/Gab1 to modulate macrophage polarization both in vitro and in vivo [13]. Gαi signaling regulates various leukocyte functions, including adhesion and chemotaxis [14, 15, 16, 17], and has also been implicated in skeleton development [18]. While these studies suggest that Gαi is involved in both immune function and bone metabolism, its specific involvement in osteoclast differentiation and osteoporosis remains unclear.

Previous studies have shown that Gαi mainly exerts its function of binding to G protein‐coupled receptors (GPCRS) and inhibiting adenylate cyclase (AC). Recent findings in our group, however, have identified broader, noncanonical functions for Gαi1 and Gαi3, particularly in mediating signaling pathways initiated by various receptor tyrosine kinases (RTKs) and other non‐RTK receptor types [11, 13, 19, 20, 21, 22, 23, 24, 25, 26, 27]. Gαi1/3 participate in receptor endocytosis and complex formation with key adaptor proteins, thereby orchestrating the robust activation of crucial intracellular signaling cascades, prominently the PI3K‐Akt‐mTOR and Erk‐MAPK pathways [11, 25, 26, 27]. This functional integration of Gαi1/3 in receptor trafficking and downstream signal transduction is essential for cellular functions [11, 25, 26, 27]. For example, Gαi1/3 are indispensable mediators of pro‐angiogenic signaling originating from several RTKs and other receptors, including VEGFR2, c‐Kit, CD146 (netrin‐1 receptor), and LGR4 (leucine rich repeat containing G protein‐coupled receptor 4, RSPO3 receptor) [11, 25, 26, 27]. The downregulation of hippocampal Gαi1/3, which were essential for BDNF‐TrkB signaling, impaired neuronal structure and induced depressive behaviors in a chronic mild stress model, a condition that could be functionally reversed by restoring Gαi1/3 expression [20, 28]. In macrophages, Gαi1/3 are essential for the LPS‐induced activation of TLR4‐CD14/Gab1 and PI3K‐Akt signaling, and their downregulation shifted macrophages toward an M2‐like phenotype, suppressing pro‐inflammatory cytokine production [13].

While previous studies suggest that Gαi is involved in both immune function and bone metabolism, its specific involvement in osteoclast differentiation and osteoporosis remains unclear. This study confirmed the crucial role of Gαi1/3 in the occurrence and development of osteoporosis and RANKL signal transduction. RANKL serves as a crucial molecular link between the bone and immune systems [29]. Given that RANK is a key regulator of osteoclast differentiation, and RANKL‐RANK signaling activates several downstream signaling pathways necessary for osteoclast function [30, 31], it remains unclear whether Gαi contributes to osteoclastogenesis through RANKL‐RANK signaling.

In this study, we conducted an exploratory research. we investigated the expression of Gαi in osteoporotic patients and osteoclast/osteoclast progenitor cells, and assessed its impact on osteoclast formation and bone metabolism. By elucidating the specific mechanisms through which the Gαi regulates bone metabolism and related signaling pathways, we identified Gαi as a crucial mediator of RANKL signal transduction. Furthermore, we demonstrated that specific functional domains of Gαi play pivotal roles in osteoclast differentiation. Notably, we found that the anti‐osteoporosis effect of denosumab was impaired in the absence of Gαi, suggesting that Gαi was integral to its therapeutic efficacy. Our findings highlighted Gαi as a promising target for osteoporosis diagnosis and treatment, paving the way for novel therapeutic strategies.

## 2 Methods

### 2.1 Approval

The human studies were approved by the Ethics Committee of the Second Affiliated Hospital of Soochow University (Approval ID: JD‐LK2022151‐IR05). All participants provided informed consent. All animal studies were reviewed and approved by the Animal Ethics Committee of Soochow University (Approval ID: SUDA20221011A10) and conducted in compliance with institutional guidelines. The mice were housed under specific pathogen‐free conditions at a controlled temperature of 22–24°C, under a 12‐h light/dark cycle, with free access to water and standard laboratory chow to ensure optimal health. All procedures were performed in accordance with international ethical guidelines and relevant national regulations [32].

### 2.2 Generation of the Gαi1/3 Double Knock (DKO) Mice

The generation of Gαi1/3 DKO mice by the CRISPR‐Cas9 method was described previously [26, 33]. The study was conducted in strict accordance with the Guide for the Care and Use of Laboratory Animals of the National Institutes of Health. All of the animals were handled according to approved institutional animal care and use committee (IACUC) protocols of Soochow University. The protocols were approved by the Committee on the Ethics of Animal Experiments of Soochow University.

### 2.3 Femoral Metaphysis Injection of AAV

The adult C57BL/6 mice (6–8 weeks old) were anesthetized, and the virus was injected into the femoral bone marrow cavity as previously described. Gαi1 or Gαi3 shRNA sequences were inserted into an adeno‐associated virus 9 (AAV9)‐Lysm construct containing the sequence of the mononuclear macrophages‐specific promoter, Lysm. AAV injection (0.1 µL virus per mouse) was performed following a previously reported protocol [26, 33].

### 2.4 LysM^Cre^; Gαi1/3^fl/fl^ Mice

*Gαi3^fl/fl^* mice were generated using the CRISPR‐Cas9 system by the Cyagen Biosciences Inc. (Suzhou, China). Two loxP sequences were inserted in the introns flanked with the exons of Gαi1/3. The *LysM^Cre^* mice were purchased from GemPharmatech Co., Ltd. *LysM^Cre^; Gαi1/3^fl/fl^* mice were generated by crossing the *LysM^Cre^* mice with *Gαi3^fl/fl^* mice for specific deletion of Gαi3 in BMMs. All experimental procedures were approved by the Soochow University's Animal Ethical Committee.

### 2.5 Establishment of Osteoporosis Model Mice

C57BL/6N mice for in vitro experiments were purchased from GemPharmatech Co., Ltd. (Nanjing, China). All the mice were kept in the specific pathogen‐free (SPF) environment at Laboratory Animal Center of Soochow University under a controlled temperature (25°C) and a 12 h day–night cycle. For OVX model, 8‐week‐old female Gαi1/3^fl/fl^ and Lysm^Cr^e; Gαi1/3^fl/fl^ mice were anesthetized with an intraperitoneal injection of pentobarbital sodium (80 mg/kg) and underwent bilateral ovariectomy or sham operation. Analgesia was achieved by a single subcutaneous injection of buprenorphine sustained‐release preparation (1 mg/kg). After 8 weeks, blood samples, femora were harvested for further study. At the conclusion of the experiment, mice were euthanized via rapid anesthesia and death induced by hypoxia in a sealed container filled with CO_2_.

The sample size was determined based on preliminary data from the evaluation of bone volume fraction (BV/TV), the primary outcome measure. Before conducting the formal experiment, we performed a preliminary exploratory study and observed a relatively consistent trend in treatment effects with low intra‐group variability. In the preliminary experiments, we observed a significant effect size (Cohen's *d* = 1.8) and a within‐group standard deviation (SD = 0.1278). Based on these findings, we performed an a priori sample size estimation using G*Power 3.1 software. Test type: Two‐sample independent *t*‐test (two‐tailed); α error probability: 0.05; statistical power (1−β): 0.80; effect size *d*: 1.8. The calculation results showed that a minimum sample size of five animals per group is required to achieve 80% statistical power. In addition, in high‐quality publications in the field of osteoporosis research using OVX‐induced osteoporotic mouse models, the typical sample size ranges from 5 to 7 mice per group [34, 35, 36]. In accordance with the “3R” principles of animal research (Reduction, Replacement, Refinement), the number of animals used should be minimized while still satisfying statistical requirements.

### 2.6 Micro‐CT and Analysis

Micro‐CT scanning and analysis were performed using the NEMO MicroCT system (Model NMC‐100). Mouse femurs were positioned between the X‐ray source and the CMOS detector and rotated 360° along the vertical axis for projection images. Images captured by the CMOS detector were processed using image analysis software with a mineralization threshold set at 350 mg/cm^3^ calcium hydroxyapatite equivalent. The scanning parameters were set to a voltage of 90 kV, a current of 0.04 mA, and a scan duration of 20 min. The volume of interest for trabecular bone analysis, located 1000 µm from the growth plate and extended a further 1500 µm longitudinally in the proximal direction region of interest (ROI). Image reconstruction was conducted with Avatar software utilizing the Feldkamp–Davis–Kress (FDK) algorithm, achieving a pixel size of 0.012 mm. Quantitative evaluations included bone mineral density (BMD), bone volume per tissue volume (BV/TV), trabecular thickness (Tb.Th), trabecular number (Tb.N), and trabecular spacing (Tb.Sp). For cortical bone, the region of analysis was 5% of femoral length in the femoral mid‐diaphysis for measuring cortical thickness (Ct.Th) [37].

### 2.7 Single‐Cell RNA Sequencing (scRNA‐seq) Data Processing

Raw sequencing data were processed using CellRanger (v4.0.0). The “cellranger mkfastq” function was used to generate FASTQ files, followed by read alignment and UMI counting using “cellranger count.” All samples were aggregated by “cellranger aggr” to normalize sequencing depth and generate the final gene expression matrix.

The expression matrix was analyzed in R (v4.4.1) using the Seurat (v4.4.0) (Integrated analysis of multimodal single‐cell data) package. Quality control was performed with SCP (https://github.com/zhanghao‐njmu/SCP). Cells were retained if they met the following criteria: 200 < nFeature_RNA < 7,500, 500 < nCount_RNA < 100,000, Mitochondrial gene percentage < 25%.

Low‐quality and doublet cells were removed. Each dataset was normalized using the “NormalizeData” function and the top 2,000 variable genes were selected by “FindVariableFeatures” (method = “vst”). Batch effects across samples were corrected using Harmony [38]. The variable genes overlapping with the list of human protein‐coding genes were used for downstream analysis.

Principal component analysis (PCA) was performed with 30 principal components, followed by UMAP for visualization (“RunUMAP”, dims = 1:30). Cells were clustered using “FindNeighbors” and “FindClusters” with a resolution of 0.5. The “IntegrateData” function was applied to merge data from all stages.

Cell identities were annotated based on canonical bone marrow markers from GeneCards and manually adjusted according to marker gene expression patterns. T cells (*Cd3d, Cd3g, Nkg7*, and *Klrd1*) were identified by the expression of pan‐T cell and cytotoxic markers. B cells and plasma cells were annotated by *Ms4a1* and *Cd19*. Mast cells were characterized by high expression of *Cpa3*, *Gata2*, *Ms4a2*, and *Hdc*. Neutrophils and other granulocytes expressed *S100a8*, *S100a9*, *Ly6g*, *Mmp8*, and *Mmp9* [39]. Osteoblasts were annotated based on the expression of *Alpl*, *Runx2*, *Col1a1*, and *Col1a2*. Osteoclasts and were identified by high expression of *Acp5*, *Ctsk*, *Tnfrsf11a*, *Nfatc1*, *Itgb3*, *Atp6v0d2*, *Spp1*, and *Oscar*, whereas osteoclast progenitors were distinguished by lower expression levels of these markers [40, 41]. MSCs were recognized by high levels of *Ly6a* and *Runx2* [42].

### 2.8 Downstream Analysis

After clustering, differentially expressed genes (DEGs) were identified using the “RunDEtest” function with thresholds of |log_2_FC| > log_2_(1.1) and adjusted *p*‐value < 0.05. Enrichment analysis was performed using the “RunEnrichment” function with GO_BP and KEGG databases (species = Mus musculus). The top 20 enriched pathways were visualized with “EnrichmentPlot.”

Osteoclast, osteoblast, and progenitor populations were analyzed separately. Expression differences of *Gnai1* and *Gnai3* were compared between control and OVX groups using “FeatureStatPlot.” UMAP visualization (“CellDimPlot”) and volcano plots (“VolcanoPlot”) were used for result presentation.

### 2.9 Genetic Modifications of Gαi1/3

As described previously [26, 33], genetic modifications in Gαi1/3 expression and function were achieved through different viral constructs. These modifications, including Gαi1/3 silencing by targeted shRNA, ectopic overexpression, and dominant‐negative (DN) mutations, performed in both BMMs. The puromycin‐containing complete medium was used to establish stable cell lines.

### 2.10 Analysis of the Bone Phenotype

Femurs were harvested from mice operated with OVX and fixed in 4 % paraformaldehyde for 24 h at 4°C. After demineralization in EDTA (10%) for 20 days, specimens were dehydrated in graded concentrations of ethanol and embedded in paraffin. The serial sections (5 µm) were obtained with a microtome for H&E and TRAP staining. For immunostaining, the sections were processed for NFATc1 (Thermo Fisher, MA3‐024). A goat‐anti‐mouse Alexa Fluor 594‐labeled secondary antibody was used for signal visualization (Abcam, ab150116). Images were acquired under a research‐grade whole‐slide scanning system (Olympus, VS200, Japan) or a confocal microscope (ZEISS, LSM 900, Germany). Histomorphometric analysis of mouse femurs was conducted following previously established protocols. In brief, osteoclasts were identified and quantified on TRAP‐stained sections, while osteoblast numbers were assessed using H&E‐stained sections.

### 2.11 Reagents

Primary antibodies against NFATc1 (MA3‐024) were provided by Thermo Fisher. Primary antibodies against RANK (ab13918) and TRAF6 (ab40675) were provided by Abcam. The other antibodies were described early [21]. Cell culture reagents, including cell culture, serum, medium, and antibiotics, were purchased from Gibco‐BRL (Suzhou, China). All primers, sequences, and viral constructs were provided by Shanghai Genechem Co. (Shanghai, China), unless otherwise mentioned.

### 2.12 qPCR, Western Blotting, and Others

Total RNA was isolated from BMMs using TRIzol reagent (ThermoFisher Scientific) according to the manufacturer's protocol. Synthesis of cDNA was performed using the PrimeScript RT Master Mix (Takara). RT‐qPCR amplification was performed with TB Green Premix Ex Taq (Tli RNaseH Plus) on a Roche LightCycler 480 II system.

Western blotting, co‐immunoprecipitation (IP) assay, and confocal microscopy were conducted as previously detailed [21, 43, 44]. For Western blotting, each SDS‐PAGE Gel lane was loaded with 40 µg of quantified protein lysates. Identical lysate samples were run in parallel on separate “sister” gels to test different proteins (same procedure for all figures).

### 2.13 Osteoclastogenesis Assay

Bone marrow cells (BMCs) from the tibia and femur were isolated and treated with red blood cell lysis buffer to remove erythrocytes. Adherent cells were cultured in minimal essential medium (α‐MEM, Gibco) with 10% FBS, macrophage colony‐stimulating factor (MCSF, R&D system), and RANKL (R&D system) in 12‐well plates after 16 h. Every two days, culture media was replaced, and cells morphology was observed for the appearance of large multinucleated cells. Cells were stained for tartrate resistant acid phosphatase (TRAP) (Sigma–Aldrich) after 5 days of culture, and the quantity of osteoclasts was counted under microscope. As for bone resorption assay, mature osteoclasts were subjected to ultrasonication after incubation in osteo assay surface (Corning) and resorption pits were visualized using light microscope.

### 2.14 TRAP Staining

TRAP staining was performed on day 6 of differentiation to evaluate osteoclast formation, unless stated otherwise. Cells were washed with PBS, fixed in 4% paraformaldehyde for 10 min, and permeabilized with 0.5% Triton X‐100 for 5 min. The TRAP staining solution contained 0.1 mg/mL naphthol AS‐MX phosphate, 10 µL/mL N, N‐dimethylformamide, and 0.6 mg/mL fast red violet LB salt in 0.1 m sodium acetate buffer (pH 5.0). Osteoclasts were identified as TRAP‐positive cells containing more than three nucleus.

### 2.15 Phalloidin Staining

BMMs seeded on glass coverslips were stimulated with MCSF and RANKL for 5–8 days. Cells were washed with PBS, fixed in 4% paraformaldehyde, permeabilized with 0.2% Triton X‐100 in PBS, and blocked with 5% goat serum. Mature osteoclasts were detected by phalloidin staining (Beyotime Biotechnology) in accordance with instructions. Fluorescent signal was observed and photographed using the Leica TCS SP6 fluorescent microscope. The number and area of actin ring was circled with dotted white line and calculated by ImageJ.

### 2.16 Statistical Analysis

Data are presented as mean ± SD. The sample size for each experiment was determined based on preliminary data or prior studies in the field to ensure adequate statistical power (typically aiming for >80% power and α = 0.05). Statistical analyses were performed using GraphPad Prism 8 software. Prior to parametric testing, the normality of data distribution was assessed using the Shapiro‐Wilk test, and the homogeneity of variances was assessed using Brown–Forsythe test or F test, as appropriate. For comparisons between two groups, unpaired two‐tailed Student's *t*‐test was used when data met the assumptions of normality and equal variance; otherwise, the nonparametric Mann–Whitney *U* test was applied. For comparisons among three or more groups, one‐way ANOVA followed by Tukey's post hoc test was used when data met the assumptions of normality and homogeneity of variances. If the assumptions for ANOVA were violated, the Kruskal–Wallis test followed by Dunn's post hoc test was employed. All experiments were repeated independently at least three times. A *P*‐value of <0.05 was considered statistically significant. Randomization and blinding were adopted to reduce bias.

## 3 Result

### 3.1 Gαi1/3 Is Involved in Osteoporosis and Osteoclast Formation

**FIGURE 1:**
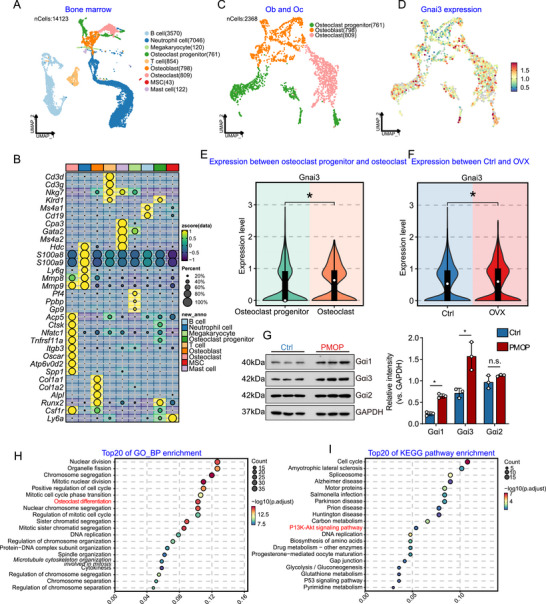
Gαi1/3 is involved in osteoporosis and osteoclast formation, signal transduction. (A) Visualization of the bone marrow scRNA‐seq data of OVX mice and sham mice using Uniform Manifold Approximation and Projection (UMAP) projections. (B) Heatmap showing normalized expression of key cell lineage marker genes. (C) UMAP of osteoclast progenitors, osteoclast, and osteoblast. (D) The expression of Gαi3 in the UMAP visualization. (E) A violin diagram of Gαi3 compares differences between osteoclast progenitors and osteoclasts. (F) A violin diagram of Gαi3 compares differences between OVX and sham mice. (G) Expression of Gαi subunits in postmenopausal osteoporosis (PMOP) patients and normal bone mass was shown. (H, I) Enrichment analysis shown possible biological process (BP) and KEGG pathways of the top 100 co‐expressing genes (CEGs) with Gαi3 in osteoclast progenitors/osteoclasts of osteoporosis model mice. *n* = 5 per group. Statistical analysis was performed using one‐way ANOVA. Data were presented as mean ± SD. *p < 0.05, n.s., no significance.

Ovariectomy (OVX) is a widely used experimental model for studying osteoporosis [45]. To examine the cellular localization of the Gαi subunit within bone marrow, we performed single‐cell sequencing on BMCs from mice. A total of 14,123 BMCs were analyzed using single‐cell sequencing. Through dimensionality reduction, clustering, and annotation, we identified nine distinct cell clusters (Figure 1A). Among these, we identified a total of 2368 cells closely associated with bone metabolism, including 761 osteoclast progenitors (characterized by low expression of *Acp5*, *Ctsk*, *Tnfrsf11a*, *Nfatc1*, *Itgb3*, *Atp6v0d2*, *Spp1*, and *Oscar*), 798 osteoclasts (characterized by low expression of *Acp5*, *Ctsk*, *Tnfrsf11a*, *Nfatc1*, *Itgb3*, *Atp6v0d2*, *Spp1*, and *Oscar*), and 809 osteoblasts (*Alpl*, *Runx2*, *Col1a1*, and *Col1a2*) (Figure 1B,C). Notably, Gαi3 expression exhibited high expression in osteoclast progenitors and osteoclasts (Figure 1D).

The violin plot demonstrated that increased Gαi3 expression in the bone marrow of OVX mice, with osteoclasts showing higher expression levels than osteoclast progenitors (Figure 1E,F). Furthermore, bone marrow was collected from osteoporosis patients, and Western blot analysis revealed an increase in the expression of Gαi1 and Gαi3, while the expression of Gαi2 remained unchanged (Figure 1G). These findings suggested that the Gαi subunit may be involved in osteoporosis and osteoclastogenesis.

We then performed an enrichment analysis of the top 100 co‐expressing genes (CEGs), which showed a significant correlation with Gαi3. The analysis highlighted the top 20 potential biological processes (BPs) associated with Gαi3, including processes relevant to angiogenesis, such as “Osteoclast differentiation,” “Organelle fission,” and “Nuclear chromosome segregation” (Figure 1H). In addition, Kyoto Encyclopedia of Genes and Genomes (KEGG) pathway analyses conducted on Gαi3 CEGs revealed the top 20 pathways (Figure 1I). Among these, several pathways were critically involved in osteoclasts formation, including “P13K‐Akt signaling pathway,” “p65 signaling pathway,” and “DNA replication” (Figure 1H). These results suggested that Gαi1/3 were involved in osteoporosis and osteoclast formation, and related signal transduction. In subsequent studies, we will explore these hypotheses in greater detail.

### 3.2 Gαi1/3 Silencing of BMMs Prevents OVX‐Induced Osteoporosis

**FIGURE 2:**
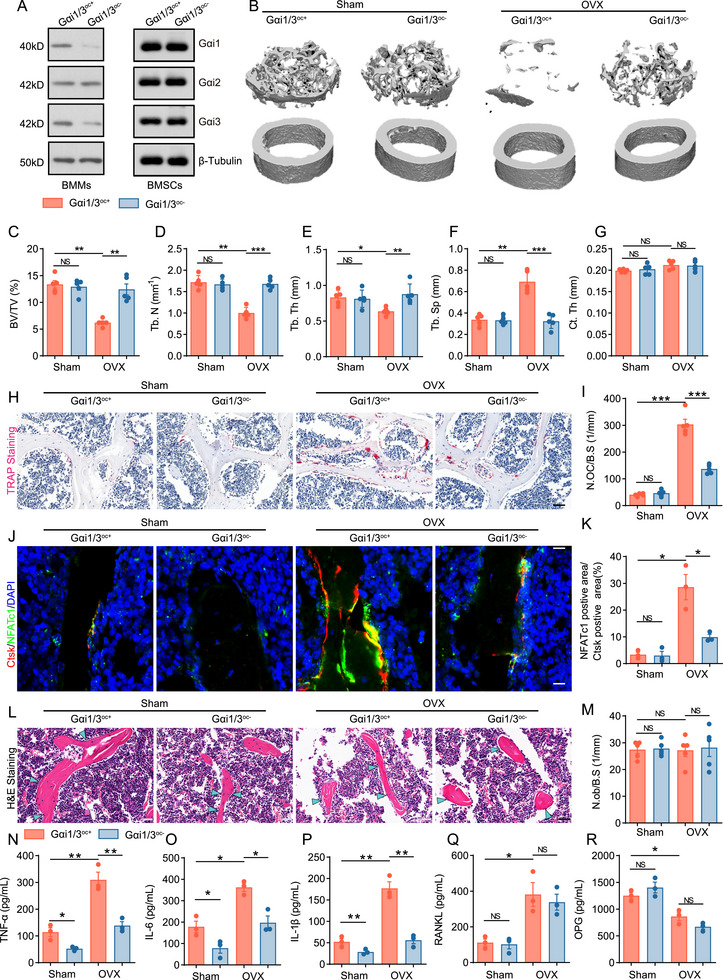
Myeloid cell‐specific deletion of Gαi1/3 attenuates OVX‐induced bone loss. (A) Western blot analysis of the relative expression levels of Gαi1, Gαi2, and Gαi3 proteins in BMMs and bone marrow mesenchymal stem cells (BMSCs) from mice in the Gαi1/3^oc+^ and Gαi1/3^oc−^ groups. (B–G) Representative µCT images and quantitative µCT analysis of cortical and trabecular bone microarchitecture in femora from mice after sham and OVX operation (BV/TV, bone volume per tissue volume; Tb.N, trabecular number; Tb.Th, trabecular thickness; Tb.Sp, trabecular separation; Ct.Th, cortical bone thickness). *n* = 5 per group. (H, I) Representative images of TRAP staining in femora after sham and OVX operation and quantification of TRAP‐positive cells in bone surface. *n* = 5 per group. Scale bar: 50 µm. (J, K) Representative images of immunofluorescence staining of femur sections from mice after sham and OVX operation and quantification of the number of NFATc1 positive cells. *n* = 5 per group. Scale bar:50 µm. (L, M) Representative images of H&E staining of femur sections from mice after sham and OVX operation and quantification of the number of osteoblasts in bone surface. *n* = 5 per group. Scale bar:50 µm. (N–Q) The concentrations of TNFα, IL‐6, IL‐1β, RANKL, and OPG in different groups of mice. *n* = 3 per group. Statistical analysis was performed using one‐way ANOVA. Data were presented as mean ± SD. NS, no significance, *p < 0.05, **p < 0.01, ***p < 0.001.

Previous single‐cell sequencing and clinical data suggested that Gαi1/3 may contribute to the development of osteoporosis. To verify this hypothesis, we generated Gαi1/3 conditional knockout mice in osteoclast precursor (Gαi1/3^oc−^). Following a previously described protocol [26, 27], AAV9‐LysM‐Gαi1 shRNA and AAV9‐LysM‐Gαi3 shRNA (5 µL) were injected into the femoral bone marrow cavity of C57BL/6J mice, generating Gαi1/3^oc−^ mice after three weeks. As a control, mice were injected with AAV9‐LysM‐scramble shRNA. The injections resulted in efficient deletion of Gαi1 and Gαi3 in BMMs and their differentiated osteoclasts, confirmed by a substantial reduction in Gαi1 and Gαi3 protein levels in BMMs from Gαi1/3^oc−^ mice, while Gαi2 expression remained unchanged. Meanwhile, the viral injection did not induce knockdown of Gαi1‐3 expression in bMSCs (Figure 2A).

OVX was performed on Gαi1/3^oc−^ and control mice (Gαi1/3^oc+^) to establish a mouse model of osteoporosis. After six weeks, bone mass in the femoral metaphysis was analyzed using Micro‐CT. The 3D reconstruction images demonstrated that bone loss was induced after ovariectomy, but this process was delayed following the knockdown of Gαi1/3 (Figure 2B). In addition, the bone morphology‐related parameters BV/TV, Tb.Th, and Tb.N were elevated, while Tb.Sp decreased in Gαi1/3^oc−^ OVX mice compared to Gαi1/3^oc+^ OVX mice (Figure 2C–G). Histological examination of femurs revealed that the number of TRAP‐positive osteoclasts on the bone surface increased in OVX mice, whereas this increase was inhibited in Gαi1/3^oc+^ mice (Figure 2H,I). Immunofluorescence staining of OVX bone tissues revealed a reduction in osteoclastic NFATc1 expression in Gαi1/3^oc−^ mice compared to Gαi1/3^oc+^ mice (Figure 2J,K). H&E staining showed that BMM‐specific knockdown of Gαi1/3 does not affect osteoblast number (Figure 2L,M).

The alterations in inflammatory cytokine levels within the marrow cavity were evaluated by ELISA. The results demonstrated that the deletion of Gαi1/3 inhibits the expression of inflammatory factors in the bone marrow (Figure 2N–P). This result is consistent with previous research findings: the absence of Gαi inhibits inflammatory responses [13]. The results showed that the concentration of RANKL increased in OVX mice, while the concentration of OPG slightly decreased. The absence of Gαi1/3 failed to alter this trend of change (Figure 2Q,R).

To further investigate the role of Gαi1/3 in osteoclastogenesis in vivo, *LysM^Cre^; Gαi1/3^fl/fl^* mice were generated by crossing the *LysM^Cre^* mice with *Gαi3^fl/fl^* mice for specific deletion of Gαi1/3 in BMMs (Figure S1A). The results of western blotting showed that Gαi1/3 in BMMs *of LysM^Cre^; Gαi1/3^fl/fl^* mice decreased, while the expression of Gαi2 did not change significantly (Figure S1B). Bone mass loss was not observed in *LysM^Cre^; Gαi1/3^fl/fl^* mice following ovariectomy (Figure S1C). Consistent with the above results, the values of BMD, BV/TV, Tb.Th, and Tb.N were all increased, while Tb.Sp decreased in *LysM^Cre^; Gαi1/3^fl/fl^* mice than *Gαi1/3^fl/fl^* mice (Figure S1D–I). Bone histomorphometric analysis of the femur indicated that the trabecular osteoclast numbers were significantly lower in *LysM^Cre^; Gαi1/3^fl/fl^* mice than *Gαi1/3^fl/fl^* mice after OVX (Figure S1J,K). This result suggests that the absence of Gαi1/3 in BMMs can inhibit the formation of osteoclasts and bone loss in osteoporosis model mice.

### 3.3 RANKL‐Induced Osteoclast Development and Function Were Abolished in BMMs Derived from Gαi1/3‐DKO Mice

**FIGURE 3:**
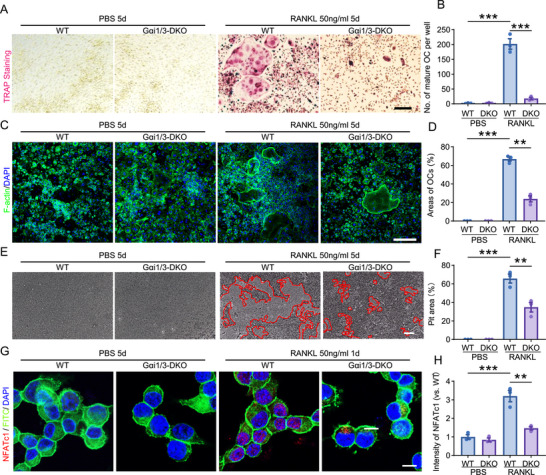
Gαi1 and Gαi3 double knockout largely inhibits RANKL‐induced osteoclastic differentiation in BMMs. (A, B) BMMs isolated from WT and DKO mice. The cells were cultured in α‐MEM medium supplemented with PBS and RANKL (50 ng/mL). On day 5, osteoclasts were stained with TRAP. Representative images of TRAP staining and quantification of the number of osteoclasts. *n* = 3 per group. Scale bar: 50 µm. (C, D) BMMs in different groups were cultured on Osteo Assay surface for 5 days in α‐MEM medium supplemented with PBS and RANKL (50 ng/mL). On day 5, osteoclasts were stained with phalloidin. Representative images of fluorescence of cell nuclei and F‐actin rings. green, actin ring; blue, DAPI. Quantification of percentage of osteoclast area. *n* = 3 per group. Scale bar: 50 µm. (E–G) BMMs in different groups were cultured on Osteo Assay surface for 5 days in α‐MEM medium supplemented with PBS and RANKL (50 ng/mL). Representative images of resorption pits on Osseo Assay surface and quantification of the resorption pit area. *n* = 3 per group. Scale bar: 100 µm. (G, H) The cells were cultured in α‐MEM medium supplemented with PBS and RANKL (50 ng/mL). On day 1, cells were stained with NFATc1. Representative images of fluorescence of NFATc1. Green, FITC; red, NFATc1. Quantification of fluorescence intensity of NFATc1 in BMMs. *n* = 3 per group. Scale bar: 50 µm. Statistical analysis was performed using one‐way ANOVA. Data were presented as mean ± SD. **p < 0.01, ***p <0.001.

RANKL is an essential and central regulator of osteoclast development and function [30]. BMMs from wild‐type (WT) and Gαi1/3 double knockout (Gαi1/3‐DKO) mice were used to investigate the role of Gαi1/3 proteins in RANKL‐induced osteoclastogenesis, as previously reported [20, 21, 26]. Next, BMMs were differentiated into osteoclasts by adding RANKL, with or without Gαi1 and Gαi3. TRAP staining assays were performed 6 days after the induction of osteoclast differentiation. RANKL induced BMMs to differentiate into osteoclasts, but the number of osteoclasts derived from BMMs of Gαi1/3‐DKO mice was significantly reduced compared to those from WT mice (Figure 3A,B). Consistent with the results above, a marked decrease in the size of the actin ring structure was observed, and the area of osteoclasts (OCs) were reduced as seen via phalloidin staining in Gαi1/3‐DKO osteoclasts (Figure 3C,D). Bone resorption assays were conducted to assess osteoclast function. Osteoclasts derived from Gαi1/3‐DKO BMMs exhibited reduced bone resorption capacity compared to WT BMMs, as manifested by decreased resorption pits and trails on the osteo surface (Figure 3E,F).

NFATc1 (nuclear factor of activated T‐cells 1), a master regulator of osteoclast differentiation, regulates several osteoclast‐specific genes, including TRAP, cathepsin K, calcitonin receptor, and osteoclast‐associated receptor (OSCAR) [46, 47]. BMMs from mice were isolated and treated with RANKL for 1 h. The expression of NFATc1 was examined by immunofluorescence. The results showed that the absence of Gαi1/3 inhibited NFATc1 expression in BMMs (Figure 3G,H).

To further investigate the effect of Gαi1/3 on osteoclast formation and function, Gαi1 and Gαi3 were knocked down in BMMs. Gαi1 shRNA and Gαi3 shRNA‐lentiviral particles, as reported previously [11, 20, 27], were transfected into BMMs. After puromycin selection, stable BMMs were established, referred to as “shGαi1/3” BMMs. In shC BMMs, RANKL treatment robustly promoted osteoclast formation. Whereas, in BMMs with Gαi1 and Gαi3 silenced (shGαi1/3 BMMs), RANKL‐induced osteoclast development was almost completely abolished (Figure S2A–D). In addition, the silencing of Gαi1/3 inhibited absorption of bone tissue and the expression of NFATc1 in BMMs (Figure S2E–H). It also hindered the expression of Ctsk, Mmp9, and Dc‐stamp (Figure S2I–K), which are closely related to osteoclast formation [48, 49]. Collectively, these results demonstrated that Gαi1/3 plays a key role in RANKL‐induced osteoclast formation and function. Gαi1/3 may be a critical protein in the treatment and prevention of osteoporosis‐related diseases.

### 3.4 Gαi1 and Gαi3 Overexpression Enhanced RANKL‐Induced Osteoclast Development, Bone Resorption Function

**FIGURE 4:**
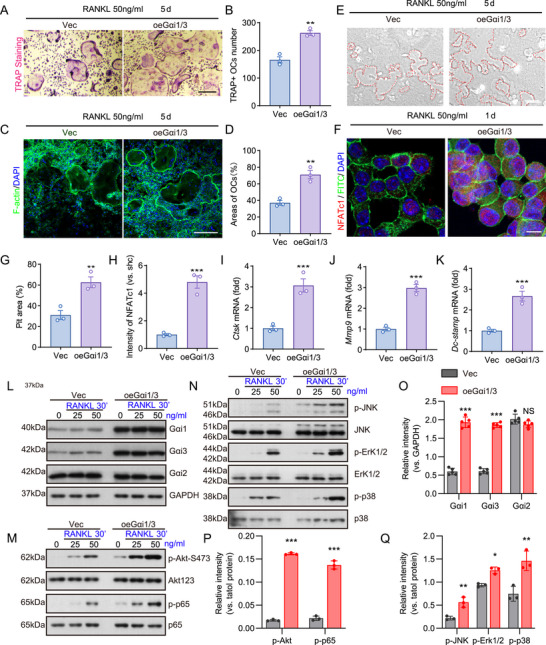
Gαi1/3 overexpression promotes RANKL‐induced osteoclast generation. (A, B) BMMs were transfected with the adenovirus Gαi1 construct and adenovirus Gαi3 construct (oe‐Gαi1/3) or the empty vector. The cells were cultured in α‐MEM medium supplemented with RANKL (50 ng/mL). On day 5, osteoclasts were stained with TRAP. Quantification of osteoclast number. *n* = 3 per group. Scale bar: 50 µm. (C, D) Osteoclasts were stained with phalloidin. Representative images of fluorescence of cell nuclei and F‐actin rings. green, actin ring; blue, DAPI. Quantification of percentage of osteoclast area. *n* = 3 per group. Scale bar: 50 µm. (E) Representative images of resorption pits on Osseo Assay surface. *n* = 3 per group. Scale bar: 50 µm. (F) BMMs in different groups were treated with RANKL (50 ng/mL). On day 1, osteoclasts were stained with NFATc1. Representative images of fluorescence of NFATc1. Green, FITC; red, NFATc1. *n* = 3 per group. Scale bar: 10 µm. (G) Quantification of resorption pit area of “E”. (H) Quantification of fluorescence intensity of NFATc1 in BMMs of “F”. (I–K) qPCR analysis of the mRNA expression of osteoclast‐specific genes in BMMs cultured in different groups with RANKL. *n* = 3 per group. (L–Q) BMMs in different groups were cultured, the proteins listed were examined by Western blotting and phosphorylation (vs total proteins) was quantified. *n* = 3 per group. Statistical analysis was performed using one‐way ANOVA. Data were presented as mean ± SD. *p < 0.05, **p < 0.01, ***p < 0.001.

Since Gαi1/3 knockdown or knockout significantly inhibited osteoclast development, function, and RANKL‐induced signaling, we hypothesized that overexpressing Gαi1 and Gαi3 would have the opposite effect, enhancing osteoclastogenesis and bone resorption function. To test this, lentiviral particles carrying Gαi1 and Gαi3‐expressing vectors were co‐transfected into BMMs, followed by puromycin selection to establish stable oeGαi1/3 cell lines. Overexpression of Gαi1 and Gαi3 promoted polykaryotic osteoclast formation. Both the number of TRAP‐positive cells and the area of osteoclasts were increased (Figure 4A–D).

Furthermore, Gαi1/3 overexpression enhanced osteoclast bone resorption capacity, resulting in larger resorption pit areas on the bone plate. In addition, increased NFATc1 expression was detected in oeGαi1/3 BMMs following RANKL stimulation (Figure 4E–H). Overexpression of Gαi1/3 also significantly upregulated the expression of key osteoclast‐associated genes, including Ctsk, Mmp9, and Dc‐stamp in BMMs (Figure 4I–K).

Given that Gαi1/3 overexpression enhanced RANKL‐induced osteoclast formation and function, we further examined its effects on molecular signaling pathways in BMMs As expected, oeGαi1/3 BMMs displayed robust increases in Gαi1 and Gαi3 expression, while Gαi2 expression remained unchanged (Figure 4L,O), compared to vector control BMMs. Moreover, RANKL‐induced phosphorylation of p65, Akt‐S473, JNK, Erk1/2, and p38 was significantly augmented in oeGαi1/3 BMMs (Figure 4O–Q). These findings were consistent with those observed in Gαi1/3 knockdown or knockout models, collectively reinforcing the crucial role of Gαi1/3 in RANKL‐induced osteoclastogenesis and signal transduction.

### 3.5 Gαi1/3 Is a Key Protein Regulator of RANKL Signal Transduction

**FIGURE 5:**
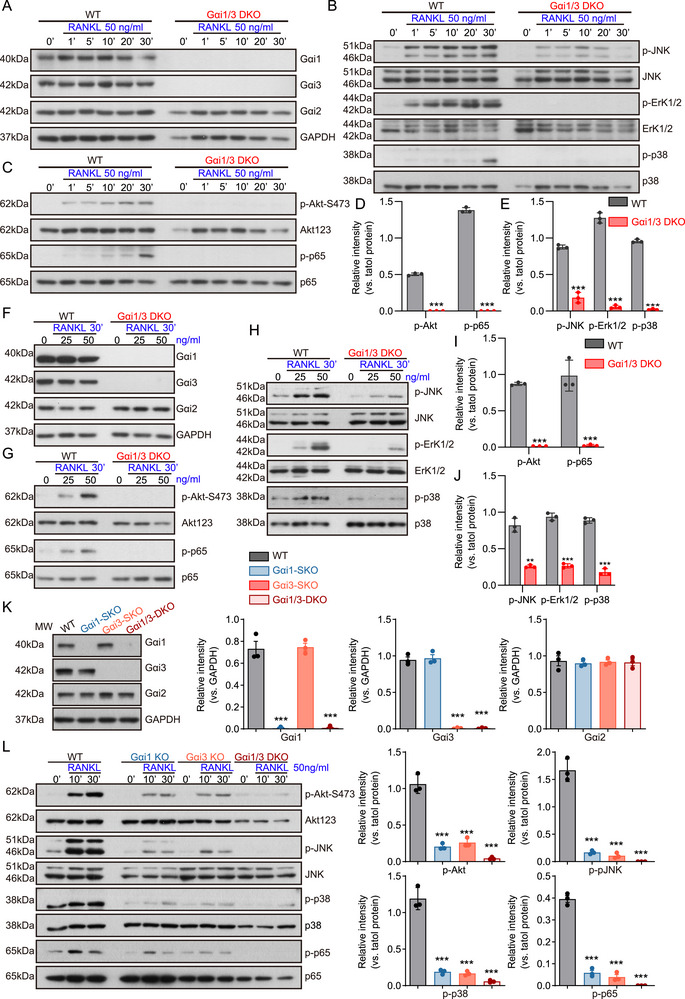
Gαi1 and Gαi3 double knockout largely inhibits RANKL‐induced downstream signal activation in BMMs. (A–E) BMMs isolated from WT and DKO mice were cultured in α‐MEM medium supplemented with RANKL (50 ng/mL) for indicated time, the proteins listed in total cell lysates were examined by Western blotting and phosphorylation (vs total proteins) was quantified. (F–J) BMMs in different groups were cultured in α‐MEM medium supplemented with RANKL (25 or 50 ng/mL) for 30 min, the proteins listed were examined by Western blotting and phosphorylation (vs total proteins) was quantified. (K) BMMs with Gαi1 or Gαi3 CRISPR/Cas9 KO constructs were cultured in α‐MEM medium, Gαi1, Gαi2, Gαi3 were examined by Western blotting. (L) BMMs with Gαi1 or Gαi3 CRISPR/Cas9 KO constructs were cultured in α‐MEM medium supplemented with RANKL (50 ng/mL) for indicated time, the proteins listed were examined by Western blotting and phosphorylation (vs total proteins) was quantified. Statistical analysis was performed using one‐way ANOVA. Data were presented as mean ± SD. ***P < 0.001.

Previous studies have demonstrated that RANKL binding to RANK on bone marrow monocytes triggers osteoclastogenesis and promotes bone resorption by activating downstream pathways such as NF‐κB and MAPK [50, 51]. To explore the role of Gαi1/3 in RANKL‐induced signaling, BMMs from Gαi1/3 DKO mice were used, as reported previously [21, 26, 27]. Western blot analysis confirmed that Gαi1 and Gαi3 were undetectable in these cells (Figure 5A).

To assess RANKL‐mediated signaling, BMMs were treated with RANKL (50 ng/mL) for 1, 5, 10, 20, or 60 min, and the activation of intracellular signaling proteins was analyzed. In WT BMMs, RANKL robustly increased the phosphorylation of p65 (Ser‐2448), Akt‐S473, JNK, Erk1/2, and p38, indicating that RANKL activates intracellular NF‐κB, Akt, and MAPK signaling. However, in Gαi1/3‐DKO BMMs, RANKL‐activated NF‐κB, Akt, and MAPK signaling were blocked at all tested time points (Figure 5B–E). Among the tested time points, 30 min of RANKL treatment elicited the strongest activation of these pathways in WT BMMs (Figure 5B–E).

To further examine the effect of RANKL concentration, BMMs were treated with increasing concentrations of RANKL for 30 min. In WT BMMs, phosphorylation of p65 (Ser‐2448), Akt‐S473, JNK, Erk1/2, and p38 increased in a dose‐dependent manner, demonstrating activation of the NF‐κB, Akt, and MAPK cascades (Figure 5F–H). Remarkably, RANKL‐induced activation of NF‐κB, Akt, and MAPK cascades in BMMs was nullified by Gαi1/3‐DKO (Figure 5F–J). The total levels of p65 (Ser‐2448), Akt‐S473, JNK, Erk1/2, and p38 were equivalent in these BMMs (Figure 5I,J).

The assessment of the individual roles of Gαi1 or Gαi3 in RANKL‐induced signaling showed that Gαi1 single knockout (SKO) or Gαi3 SKO in BMMs resulted in only a partial reduction of p65, Akt‐S473, JNK, and Erk1/2 phosphorylation by RANKL (Figure 5K,L). Only in Gαi1/3‐DKO BMMs was RANKL‐activated signaling completely blocked (Figure 5K,L). To further validate these findings, we examined whether RANKL‐activated signaling was suppressed. RANKL‐induced activation of downstream signaling was largely inhibited following Gαi1/3 knockdown in BMMs (shGαi1/3). Phosphorylation of p65, Akt‐S473, JNK, Erk1/2, and p38 by RANKL was almost completely blocked in shGαi1/3 BMMs (Figure S3A–F). Taken together, these results demonstrated that Gαi1/3 is a critical regulator of RANKL‐mediated signal transduction, playing an essential role in NF‐κB, Akt, and MAPK pathway activation during osteoclastogenesis.

The deficiency of estrogen after menopause can relieve the inhibition of RANKL through multiple molecular pathways, thereby significantly increasing the expression of RANKL. Estrogen withdrawal makes osteoblasts, activated T/B lymphocytes and bone marrow stromal cells the main sources of excessive RANKL production. Together, they create a high‐concentration RANKL environment in the local bone area, which abnormally activates osteoclasts, leading to greater bone resorption than bone formation and triggering osteoporosis.

Taking osteoblasts as an example, we co‐cultured BMMs with osteoblasts in a medium containing estrogen (E2, 1 × 10^−7^ m). To determine the effects of estrogen deficiency on Gαi1/3 expression in macrophages, we first treated BMMs cells with 100 nmol·L^−1^ 17β‐estradiol for 24 h, and estrogen was then either maintained or withdrawn from the culture medium for another 24 h. The results of western blotting showed that the expression of Gαi1/3 in BMMs significantly increased after the discontinuation of estrogen (Figure S3G–J). This is consistent with the results of Figure 1 in vivo. In the co‐culture system, the absence of estrogen facilitates the differentiation of WT BMMs into osteoclasts, but does not support the differentiation of DKO‐Gαi1/3 BMMs into osteoclasts (Figure S3K,L).

To further confirm that Gαi1 and Gαi3 functional overlapping. we performed reciprocal overexpression rescue experiments. These experiments confirmed functional redundancy between Gαi1 and Gαi3. TRAP staining results showed that in Gαi1 or Gαi3 knockdown BMMs, overexpression of either Gαi3 or Gαi1 enhanced osteoclast differentiation capacity, as evidenced by increased osteoclast area and number (Figure S4A,B). Consistent with these findings, overexpression of Gαi3 or Gαi1 in Gαi1‐shRNA or Gαi3‐shRNA BMMs upregulated the mRNA expression of osteoclast‐related genes (*Atp6v0d, Ctsk*, and *Mmp9*) (Figure S4C).

### 3.6 Gαi1/3 Mediated RANK‐TRAF6 Binding Through the 173 Asp Residue, Regulating RANKL Signaling and Osteoclastogenesis

**FIGURE 6:**
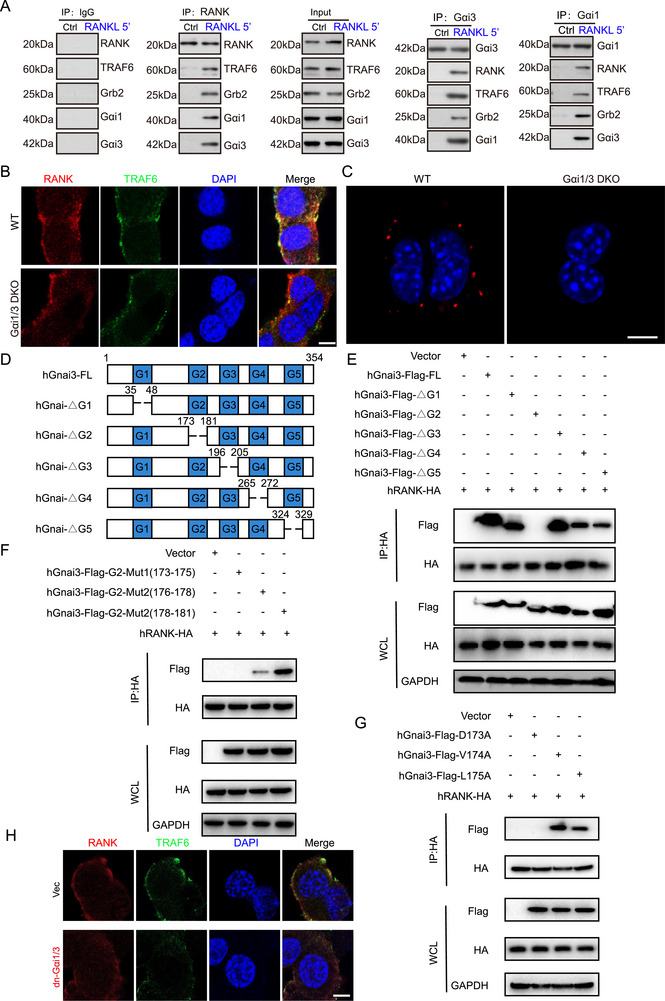
Gαi1/3 mediates RANK‐TRAF6 binding through the 173 Asp residue, regulating RANKL signaling and osteoclastogenesis. (A) BMMs were treated with RANKL (50 ng/mL) for 5 min, and the association of RANK, TRAF6, Grb2, Gαi1, and Gαi3 was examined by co‐immunoprecipitation (Co‐IP) assays. (B) BMMs isolated from WT and DKO mice were treated with RANKL (50 ng/mL) for 5 min, representative immunofluorescence images showing the role of Gαi1/3 in RANK‐TRAF6 interactions. Green, TRAF6; red, RANK. Scale bar: 10 µm. (C) BMMs isolated from WT and DKO mice were treated with RANKL (50 ng/mL) for 5 min, In situ PLA technology indicated endogenous RANK‐TRAF6 protein interactions in BMMs. Red, positive interaction complexes. Scale bar: 10 µm. (D) Schematic summary of Gnai3 and its truncation mutants, depicting the G1, G2, G3, G4, and G5 domain. (E) Co‐IP analysis of RANK interaction with Gnai3 truncation mutants using whole‐cell lysates of RAW264.7 cells transfected with the indicated expression vectors. (F) Co‐IP analysis of RANK interaction with G2 domain mutants using whole‐cell lysates of RAW264.7 cells transfected with the indicated expression vectors. (G) Co‐IP analysis of RANK interaction with point mutants using whole‐cell lysates of cells transfected with the indicated expression vectors. (H) BMMs with the dominant negative DN Gαi3‐173A construct (“dnGαi1/3”), or the vector control (“Vec”), representative immunofluorescence images showing the role of Gαi3‐173A in RANK‐TRAF6 interactions. Green, TRAF6; red, RANK. Scale bar: 10 µm.

RANK‐TRAF6 binding is essential for the activation of the RANKL signaling activation and subsequent osteoclastogenesis [30]. To determine the role of Gαi1/3 in this process, we performed a co‐immunoprecipitation (Co‐IP) assays following RANKL treatment (5 min) of BMMs and subsequent protein extraction. The results demonstrated that RANKL stimulation promoted the formation of a RANK‐Gαi1/3‐TRAF6 complex, suggesting a role for Gαi1/3 in facilitating RANK‐TRAF6 interaction (Figure 6A).

To further validate this observation, confocal microscopy of BMMs showed that, upon RANKL stimulation, membrane‐bound RANK associated with TRAF6. However, the absence of Gαi1/3 disrupted this interaction, preventing RANK‐TRAF6 binding (Figure 6B). Consistently, in situ proximity ligation assay (PLA) confirmed that the direct interaction between endogenous RANK and TRAF6 was abolished in Gαi1/3‐deficient BMMs (Figure 6C). These findings suggested that Gαi1/3 played a critical role in mediating RANK‐TRAF6 binding, thus influencing RANKL signaling and osteoclast function.

To characterize the molecular basis of Gαi1/3‐RANK interaction, we constructed a truncated mutant plasmid using Gαi3 as a model (Figure 6D). Co‐IP assays confirmed a strong interaction between Gαi3 and RANK, further revealing that the G2 domain of Gαi3 was critical for this interaction (Figure 6E). To pinpoint the specific amino acids involved, mutational analysis of the 173–175 region demonstrated that mutations in this sequence disrupted the Gαi3‐RANK interaction, indicating that this region was crucial for binding (Figure 6F).

In addition, a three‐point mutant plasmid targeting the 173–175 sequence was generated, and Co‐IP experiments revealed that the 173 Asp of Gαi3 was essential for RANK binding (Figure 6G). To functionally validate these findings, we employed dominant‐negative (dn) strategies, where dn‐Gαi1/3 constructs replaced the conserved 173‐Asp residue with Thr. WT BMMs were co‐transfected with dn‐Gαi1 and dn‐Gαi3 constructs, and confocal microscopy showed a significant reduction in RANK‐TRAF6 binding in dn‐Gαi1/3 BMMs (Figure 6H).

Importantly, RANKL‐induced osteoclastogenesis and bone resorption were abolished in dn‐Gαi1/3 BMMs, indicating a crucial role of Gαi1/3 in osteoclast differentiation and function (Figure S5A–H). In addition, dn‐Gαi1/3 significantly reduced the expression of key osteoclast‐related genes, including *Ctsk*, *Mmp9*, and *Dc‐stamp*, which were essential for osteoclast differentiation and resorptive activity (Figure S5I–K) [48, 49]. Furthermore, RANKL‐induced activation of NF‐κB, Akt, and MAPK cascades was significantly suppressed in DN‐Gαi1/3 BMMs, further confirming that Gαi1/3 played a key role in RANKL‐mediated signaling (Figure S5L–Q).

The TRAP staining results showed that the osteoclast formation capacity of DN‐Gαi3 BMMs was significantly impaired, including a notable reduction in both the osteoclast area per field and the number of osteoclasts. However, overexpression of Gαi3 in DN‐Gαi1/3 BMMs significantly enhanced their osteoclast formation capability (Figure S6A‐B). Additionally, qPCR results demonstrated that the mRNA expression of osteoclast‐related genes *Atp6v0d*, *Ctsk*, and *Mmp9* were downregulated in dn‐Gαi3 BMMs, while their expression was partially restored upon Gαi3 overexpression (Figure S6C). Western blot analysis further revealed that the protein levels of *MMP9*, *CFOS*, and *CTSK* were markedly decreased in DN‐Gαi3 BMMs, but were rescued following Gαi3 overexpression in these cells (Figure S6D).

In summary, our findings demonstrated that the 173 Asp residue of Gαi1/3 was a critical residue for mediating RANK‐TRAF6 binding. Mutation of this residue profoundly disrupts RANKL‐induced signaling, osteoclast differentiation, and bone resorption, highlighting Gαi1/3 as a pivotal regulator of osteoclastogenesis.

### 3.7 Inhibition of Gαi1/3 Exhibits Anti‐osteoporosis Effects Comparable to Denosumab Mimics

Denosumab is a clinically approved monoclonal antibody that effectively treats osteoporosis by binding to RANKL, thereby preventing its interaction with RANK on osteoclast precursor cells and inhibiting osteoclastogenesis and bone resorption [52]. However, as a humanized antibody, denosumab may elicit an immune response in mice following long‐term use [53]. To address this limitation, we employed denosumab mimics (IK22/5, 8 mg/kg, anti‐mouse RANKL antibody) to evaluate their anti‐osteoporosis effect as an alternative to denosumab.

**FIGURE 7:**
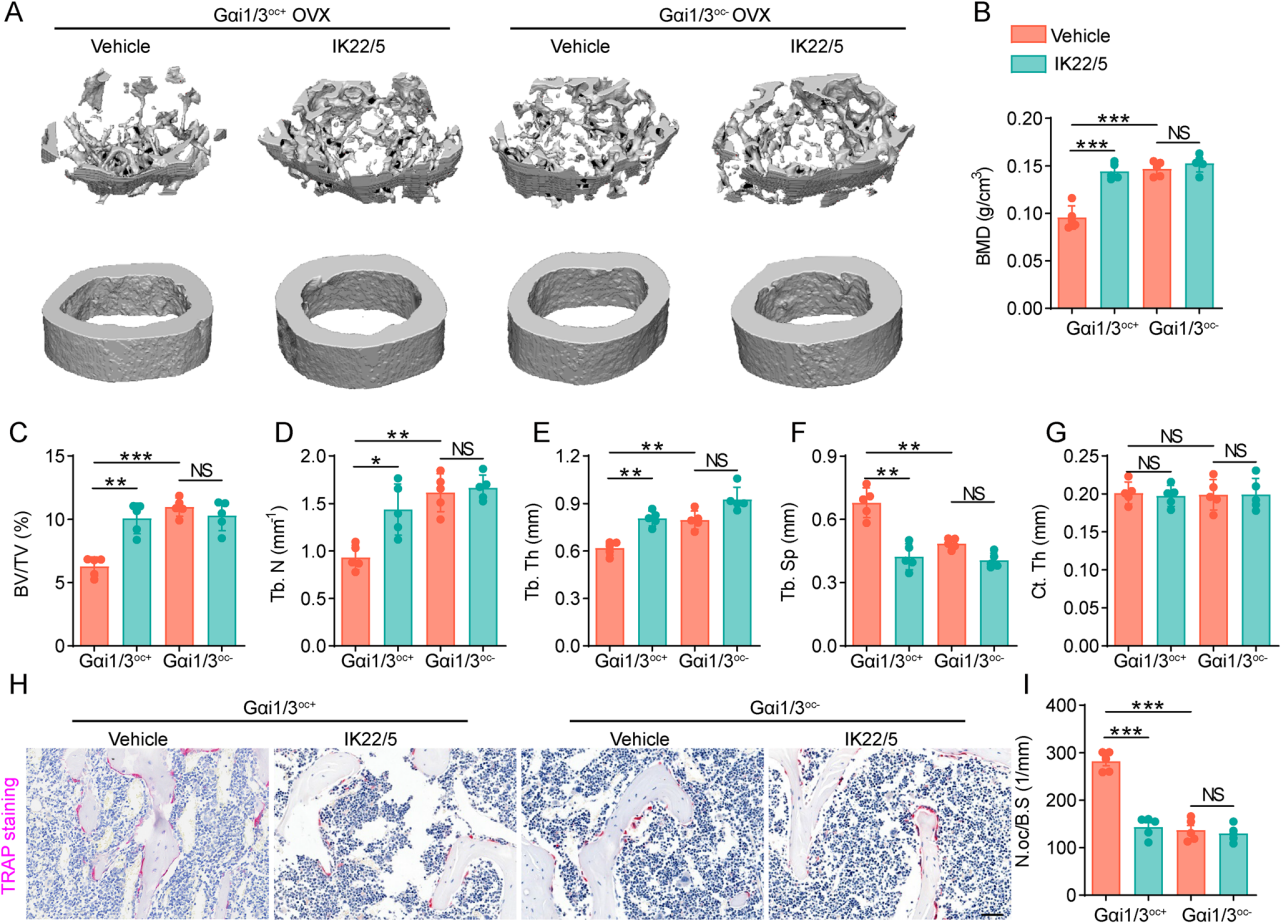
Inhibition of Gαi1/3 exhibits anti‐osteoporosis effects comparable to denosumab mimics. (A–G) Adult C57BL/6 mice (6–8 weeks old, five female in each group) received femoral metaphysis injections of either murine AAV9‐Lysm‐Gαi1/3 shRNA, generating Gαi1/3^oc−^ mice after three weeks. Control group mice were injected AAV9‐Lysm‐scramble control shRNA. Denosumab mimics (IK22/5, 8 mg/kg, anti‐mouse RANKL antibody) treatment was administered after successful establishment of the OVX model. Representative µCT images and quantitative µCT analysis of cortical and trabecular bone microarchitecture in femora from mice in the different groups (BMD, Bone Mineral Density; BV/TV, bone volume per tissue volume; Tb.N, trabecular number; Tb.Th, trabecular thickness; Tb.Sp, trabecular separation; Ct.Th, cortical bone thickness). *n* = 5 per group. (H, I) Representative images of TRAP staining in femora after sham and OVX operation, and quantification of TRAP‐positive cells in bone surface. *n* = 5 per group. Scale bar: 50 µm. Statistical analysis was performed using one‐way ANOVA. Data were presented as mean ± SD. NS, no significance, *p < 0.05, **p < 0.01, ***p < 0.001.

Osteoporosis was induced in Gαi1/3^oc+^ and Gαi1/3^oc−^ mice, which were then treated with denosumab mimics. After 6 weeks, bone mass in the femoral metaphysis was analyzed using micro‐CT. 3D reconstruction images showed that denosumab mimics delayed bone loss in Gαi1/3^oc+^ mice, and the process of bone loss was slowed after Gαi1/3 knockdown. Notably, denosumab mimics did not exert additional anti‐osteoporosis effects in Gαi1/3^oc−^ mice (Figure 7A).

Furthermore, bone morphometric parameters, including BMD, BV/TV, Tb.N, Tb.Th, and Tb.Sp were not significantly changed in the denosumab mimics group compared to the vehicle group of Gαi1/3^oc−^ OVX mice (Figure 7B–G). To further evaluate osteoclast activity, histological analysis of femurs was performed. Trap^+^ osteoclast cells on the surface of bone tissue decreased vehicle group of Gαi1/3^oc−^ OVX mice, denosumab mimics did not further reduce osteoclasts on the bone surface (Figure 7H,I). These findings suggest that Gαi1/3 inhibition has comparable efficacy to denosumab mimics in conferring anti‐osteoporosis effects.

In addition, ELISA analysis of serum from OVX mice treated with IKK22/5 revealed reduced expression of inflammatory markers, including TNF‐α, IL‐6, and IL‐1β (Figure S7A–C). Histopathological examination of the heart, liver, spleen, lungs, and kidneys from IKK2/5‐treated mice revealed no pathological changes (Figure S7D). Administration of IKK22/5 did not produce obvious side effects in mice.

## 4 Discussion

Our research highlights the pivotal roles of Gαi1 and Gαi3, key members of the Gαi protein family, in the pathogenesis of osteoporosis. A growing body of evidence has established the involvement of Gαi proteins in regulating immune cell function and the inflammatory response [12, 13, 16, 21]. Furthermore, dysregulations within the Gαi subfamily may impact bone metabolism [18, 54, 55]. However, the precise role of Gαi proteins in osteoporosis remains poorly understood. Here, we demonstrate that Gαi proteins are essential for osteoclast differentiation and bone resorption. We found that Gαi1/3 promotes the interaction between RANK and TRAF6 via the Asp173 residue, thereby regulating RANKL signaling and osteoclastogenesis. Importantly, the functional blockade of Gαi in vivo markedly delayed osteoporosis onset, with an efficacy comparable to a RANKL inhibitor.

Gαi proteins were initially categorized based on their ability to inhibit adenylyl cyclase. Epinephrine, acetylcholine, dopamine, and serotonin utilize Gαi proteins to trigger physiological responses. Upon binding with GPCRs, Gαi proteins are activated and subsequently release Gβγ subunits, which directly couple to downstream signaling pathways [56]. Previous studies have demonstrated that G proteins are crucial immunomodulatory factors regulating migration, activation, survival, proliferation, differentiation, and cytokine secretion in immune cells, including macrophages [13, 57, 58]. In our study, we observed an increase in Gαi3 expression during the differentiation of mononuclear macrophages into osteoclasts, along with significantly elevated Gαi1/3 expression in the bone marrow of osteoporosis patients. Consistently, in the OVX mouse model, osteoclast formation and bone mass loss were markedly reduced in Gαi1/3‐DKO and Gαi1/3^oc−^ mice compared to controls, thereby confirming that Gαi proteins are key regulators of osteoclast function and bone metabolism. Although single‐cell sequencing did not reveal significant changes in Gαi1 expression, differences were detected in bulk analyses of osteoporosis patients, possibly due to limitations in single‐cell resolution and computational constraints. In Figure 1, the expression of Gαi1/3 is elevated in OVX mice with estrogen deficiency and postmenopausal women. Since estrogen decline is a key driver of increased osteoclastogenesis, we conducted in vitro cell experiments to evaluate the effects of estrogen supplementation or withdrawal on Gαi1/3 expression and its impact on RANKL‐induced osteoclast formation. The in vitro experiments revealed that estrogen withdrawal promotes Gαi1/3 expression and osteoclast formation in BMMs, and the absence of Gαi1/3 eliminates the effect of estrogen on osteoclast formation (Figure S3). These results further confirm that Gαi protein is a viable therapeutic target for osteoporosis. Gαi1 and Gαi3 share 95% amino acid sequence identity and exhibit considerable structural similarity [13]. Both proteins play crucial roles in the development of osteoporosis, and it remains challenging to determine the primary contributor. This issue warrants further investigation.

RANKL is a critical molecule that links the bone and immune systems [29]. RANKL stimulation activates downstream signaling pathways of RANK, including NF‐κB and MAPK [30]. This triggers the differentiation of macrophage cells into osteoclasts. Subsequently, multinucleated osteoclasts are formed through the fusion of preosteoclasts, preparing them for bone resorption [59]. This study reveals a novel mechanism for the role of Gαi1/3 in RANKL‐mediated signal transduction that underlies osteoclast formation. We demonstrate that Gαi1/3 is required for RANKL‐induced activation of NF‐κB and MAPK. In BMDMs, human macrophages, and MEFs, Gαi1/3 KO, shRNA, CRISPR‐induced Gαi1/3 gene editing, or dominant negative Gαi1/3 mutations strongly inhibited RANKL‐induced NF‐κB and MAPK activation. Conversely, overexpression of Gαi1/3 enhanced RANKL‐induced activation of NF‐κB and MAPK. Functional studies show that BMDMs with Gαi1/3 deficiency are resistant to RANKL‐induced osteoclast formation and bone resorption, while Gαi1/3‐overexpressing BMDMs exhibit enhanced osteoclast fusion and bone resorption in response to RANKL. Our findings support that Gαi1/3 is essential for RANKL‐RANK signaling, osteoclastogenesis, and bone resorption. RANKL is a critical central regulator of the differentiation of mononuclear macrophages into osteoclasts [60]. M‐CSF is a key cytokine that induces RANK expression on the cell membrane of mononuclear macrophages and osteoclast precursors. Osteoclast progenitors expressing RANK bind to RANKL, thereby inducing osteoclast differentiation [61, 62].

Although Gαi1/3 is crucial for osteoclast differentiation and RANKL signaling, the mechanisms underlying these roles remain unclear. Previous studies show Gαi1/3 interacts with receptors CD146 and TrkB to activate downstream signaling [28, 63]. Our study confirm that RANKL promotes Gαi1/3 binding to RANK‐TRAF6, and loss of Gαi1/3 prevents this complex formation, obstructing downstream signaling [64, 65]. Gαi1/3 plays a key role in RANK‐TRAF6 binding, with inhibitory Gα subunits sharing 85%–95% sequence identity and overlapping expression patterns. We perform mutagenesis on Gαi3 and found that Asp173 is crucial for mediating the interaction with RANK. Moreover, a dominant‐negative mutation at Asp173 (DN‐Gαi1/3) inhibits RANK‐TRAF6 binding, thereby suppressing RANKL‐induced signaling and osteoclast differentiation. This enhances our understanding of Gαi1/3 in osteoporosis and paves the way for developing anti‐osteoporosis therapies. Although the in vivo significance of Asp173 in this context remains unverified, future studies should address this limitation and explore the potential of small molecules targeting Gαi1/3 for osteoporosis treatment.

Our research confirmed that RANKL‐induced osteoclast differentiation and osteoporosis require the mediation of Gαi1/3. Currently, denosumab, a humanized monoclonal antibody targeting RANKL, is a commonly used antiresorptive treatment for osteoporosis [66]. Our results suggest that denosumab mimics have a stronger anti‐osteoporosis effect in mice with normal osteoclast‐specific expression of Gαi1/3 (Gαi1/3^oc+^). In contrast, osteoporosis was inhibited in Gαi1/3^oc−^ mice, the additional anti‐osteoporotic effect of denosumab mimics was not observed. These findings imply that targeted inhibition of Gαi1/3 may be as effective as denosumab in treating osteoporosis. Despite its efficacy, denosumab discontinuation rapidly activates osteoclasts, accelerating bone loss and increasing fracture risk [67, 68]. Older, dialysis‐dependent female patients are prone to hypocalcemia after denosumab treatment [69]. These limitations restrict denosumab's widespread use. Targeting Gαi1/3 may have the same anti‐osteoporosis effect. Whether targeting Gαi1/3 can lead to more effective anti‐osteoporosis treatment and fewer side effects requires further in‐depth research in subsequent work.

So far, no studies have reported on the relationship between Gαi1/3 and osteoclasts. Neither have any clinically available or investigational drugs been found to directly target Gαi1/3. However, certain indirect agonists and inhibitors that modulate Gαi protein activity do exist. For example, pertussis toxin inactivates Gαi via ADP‐ribosylation, leading to irreversible blockade of the Gαi signaling pathway [70]. Opioid receptor agonists (such as morphine, fentanyl, and tramadol) inhibit adenylate cyclase (AC), reduce cAMP levels, and activate GIRK channels, resulting in cell hyperpolarization and decreased release of neurotransmitters (e.g., glutamate and substance P), thereby blocking pain signal transmission [71]. Nonetheless, none of these agents act directly on Gαi1/3, and their potential therapeutic effects on osteoporosis remain difficult to assess.

Our finding clarifies the mechanism of RANKL signaling by identifying Gαi1/3 as essential intracellular mediators. While this raises the theoretical question of their therapeutic potential, significant hurdles exist. These include the lack of specific drugs, the challenge of intracellular delivery, and the high risk of off‐target effects due to the ubiquitous nature of Gαi proteins. Specifically, Gαi proteins are ubiquitously expressed and are fundamental to countless signaling pathways in nearly all cell types. Systemic inhibition of Gαi1/3 would be predicted to cause widespread and severe off‐target effects. Our finding in Figure 7—that a denosumab analog provided no additional anti‐osteoporotic effect in Gαi1/3 KO mice—strongly implies that Gαi1/3 are directly downstream of, and essential for, the RANKL signal. Thus, targeting this pathway would be mechanistically redundant to existing RANKL inhibitors. Building on this research, we now aim to develop therapeutic drugs that target Gαi1/3 in osteoclasts, to achieve effective and safe treatment of osteoporosis.

Although we found that the combination of Gαi1/3 inhibitors and denosumab might enhance the efficacy against osteoporosis. Studies on denosumab effects typically require huRANKL transgenic mice [72]. In the absence of such models, we utilized mouse‐specific RANKL antibodies as a surrogate for denosumab, which may not fully recapitulate the clinical anti‐osteoporotic effects of denosumab. More effective and precise research still needs to be further carried out in the future.

This study highlights the need for further exploration of the long‐term anti‐osteoporosis effects of Gαi1/3 inhibition and potential side effects. Conducted only in mice, this study's findings cannot be directly extended to primates, and clinical translation remains distant. However, the primary contribution is the identification of a new target for anti‐osteoporosis drug development, which could improve osteoporosis diagnosis and treatment.

Single‐cell RNA sequencing and experiments revealed elevated Gαi subunit levels in osteoclasts and bone marrow of osteoporosis patients. The absence of Gαi1/3 significantly inhibits osteoclast differentiation and bone resorption. We elucidate how Gαi1/3 mediates osteoporosis by facilitating RANK‐TRAF6 binding. Notably, the Asp173 residue in Gαi1/3 was identified as critical for its interaction with RANK. These findings may guide the development of future anti‐osteoporosis drugs. Ultimately, Gαi1/3 emerges as a promising therapeutic target for clinical applications.

## 5 Conclusion

Our study provides compelling evidence that Gαi1 and Gαi3 are critical regulators of osteoclast differentiation and bone resorption in osteoporosis. Using both in vitro and in vivo models, we demonstrated that the absence of Gαi1/3 disrupts RANKL‐induced NF‐κB and MAPK activation by impairing RANK–TRAF6 complex formation, with Asp173 identified as a key residue mediating this interaction. These findings advance our understanding of the molecular mechanisms underlying osteoporosis and highlight the potential of targeting Gαi1/3 as an alternative therapeutic strategy to existing treatments such as denosumab. Although limited by model systems and sample size, our results provide valuable insights for future studies aimed at validating these mechanisms in clinical settings and guiding policy decisions in osteoporosis management.

## Author Contributions

S.C., X.Z., C.C., and H.S. conceptualized the study. All listed authors performed the experiments and the statistical analysis. C.B. and M.Z. wrote original draft.

## Conflicts of Interest

The authors declare no conflict of interest.

## Supporting information




**Supporting File 1**: advs74185‐sup‐0001‐FigureS1‐S7.pdf.

**Supporting File 2**: advs74185‐sup‐0002‐FigureCaptions.docx.

**Supporting File 3**: advs74185‐sup‐0003‐Antibodies.docx.

**Supporting File 4**: advs74185‐sup‐0004‐ImageofBlots.pdf.

## Data Availability

The data that support the findings of this study are available from the corresponding author upon reasonable request.
