# Supplementary figures and images for: Gαi1/3 Is a Novel Regulatory Target for RANKL Signal Transduction and Osteoporosis
Source: Adv Sci (Weinh). 2026 Feb 12;13(20):e10836. doi: 10.1002/advs.202510836 (PMC13067840; doi:10.1002/advs.202510836)

FIG S1

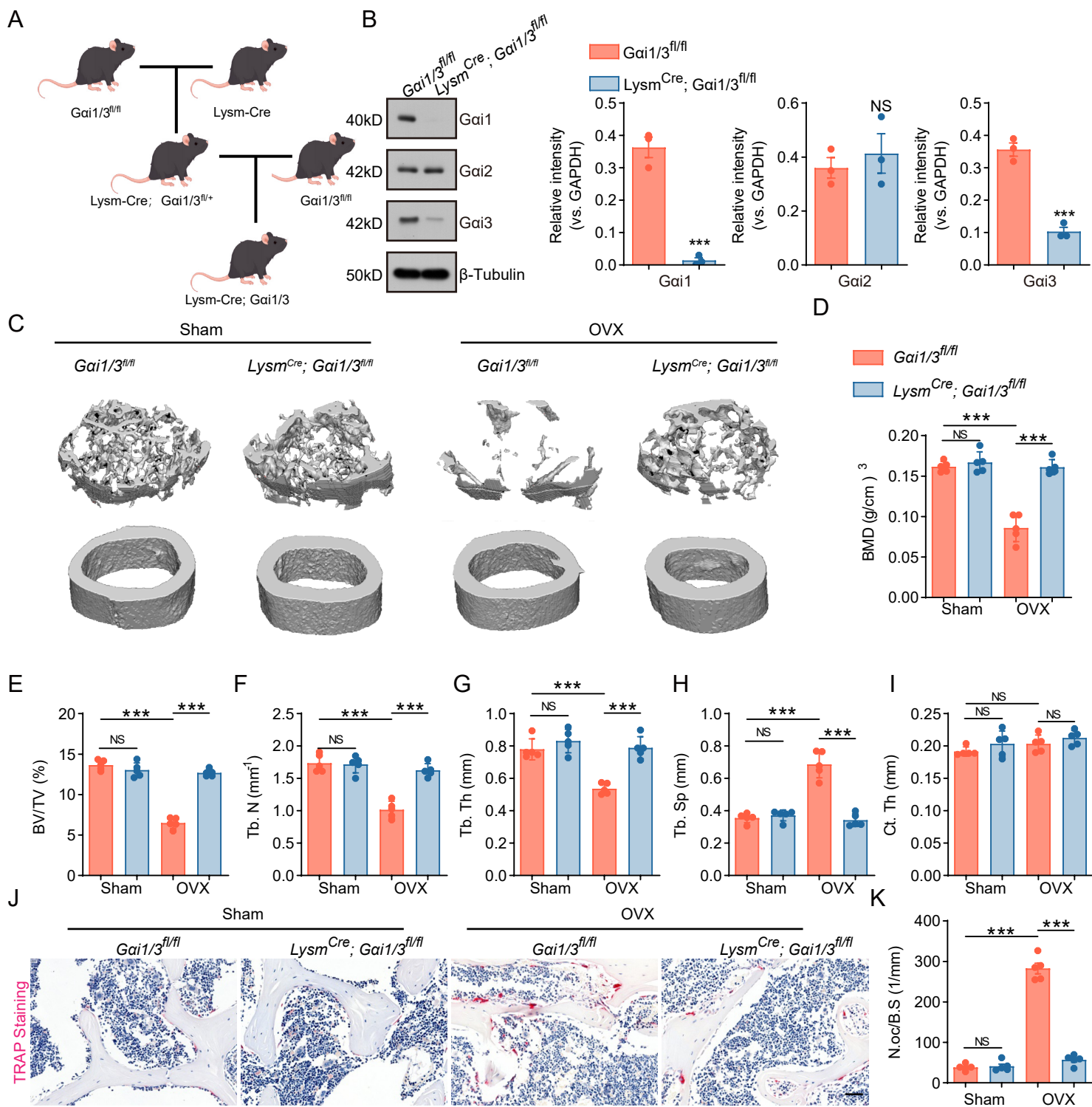

FIG S2

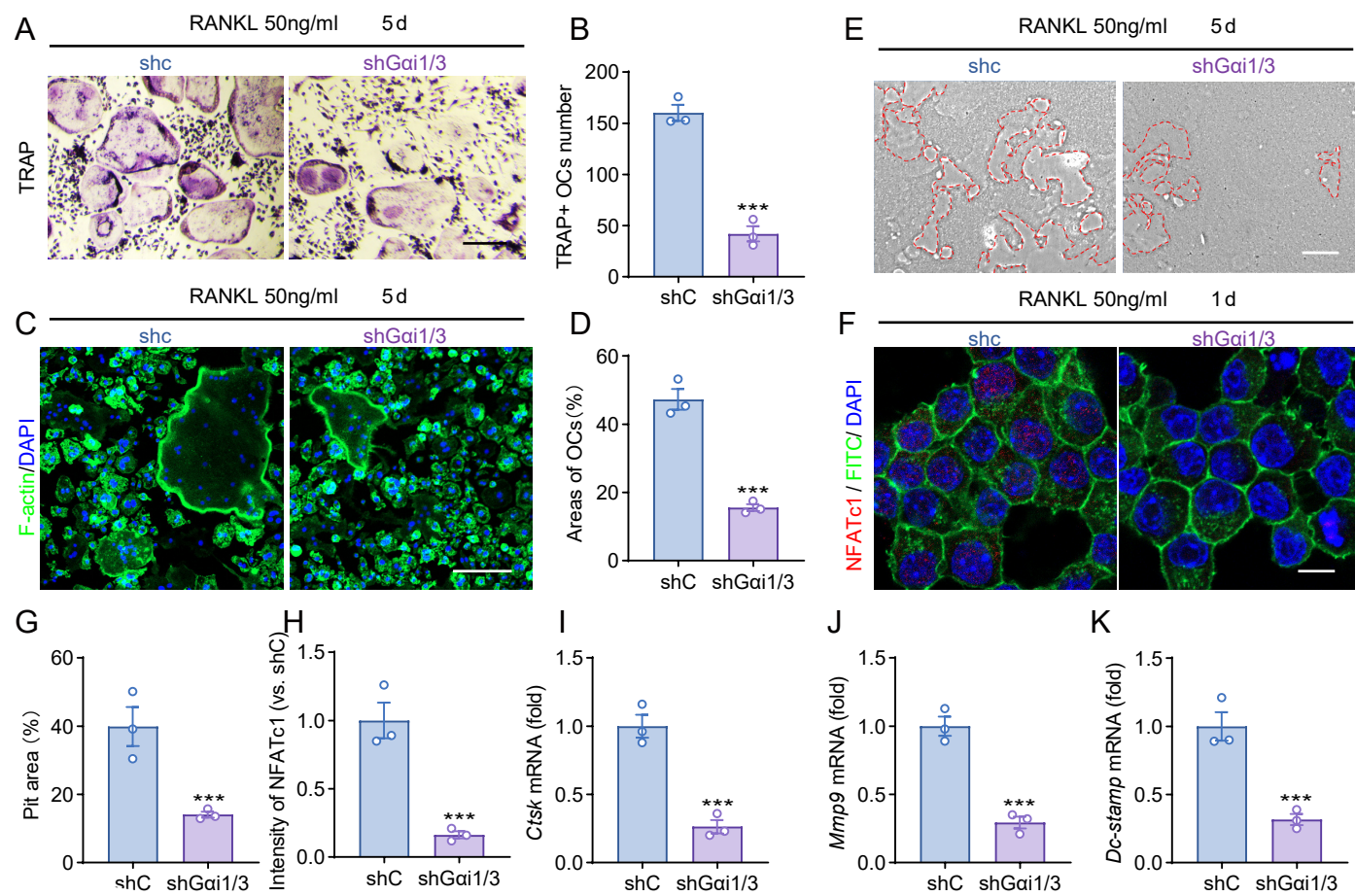

FIG S3

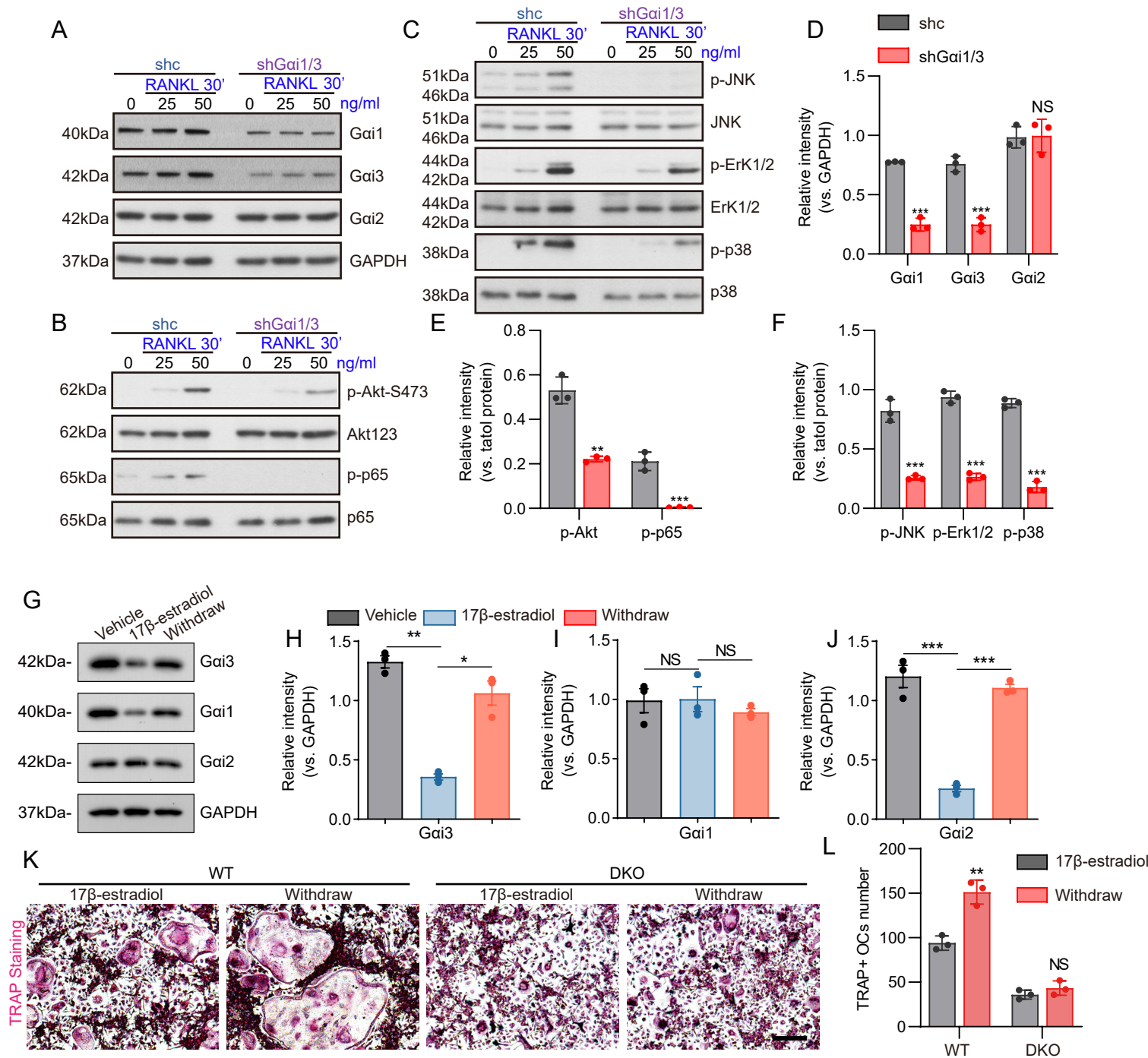

FIG S4

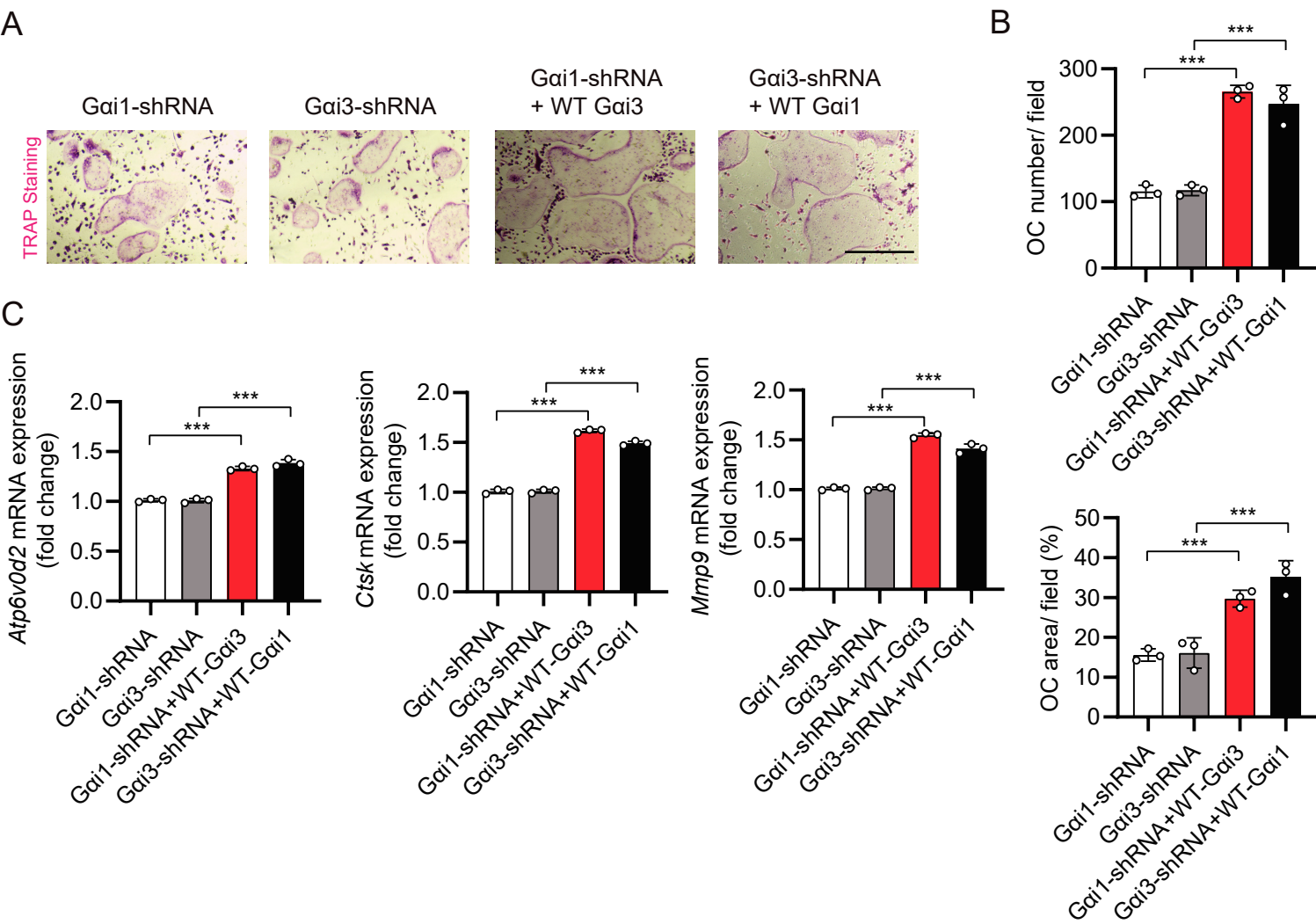

FIG S5

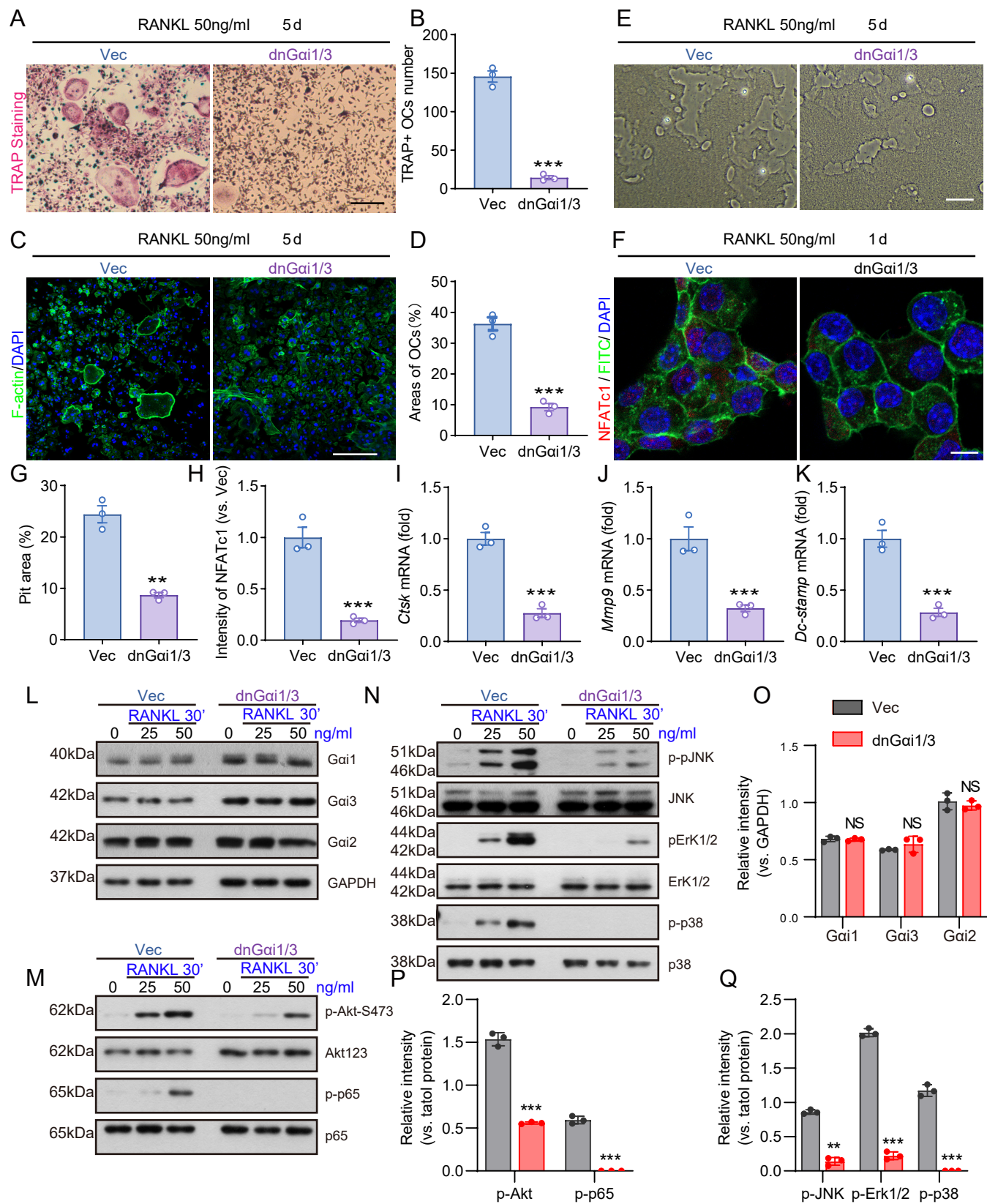

FIG S6

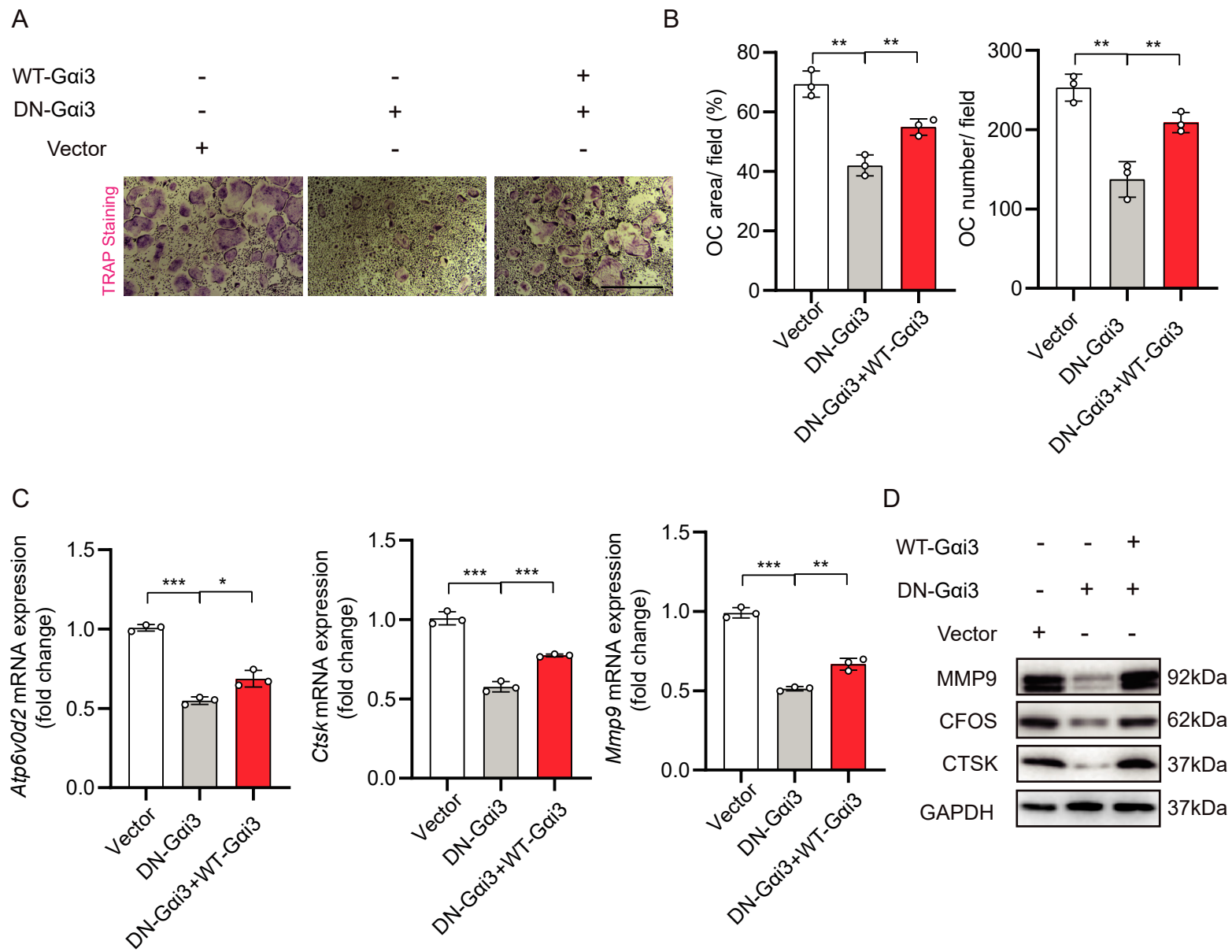

FIG S7

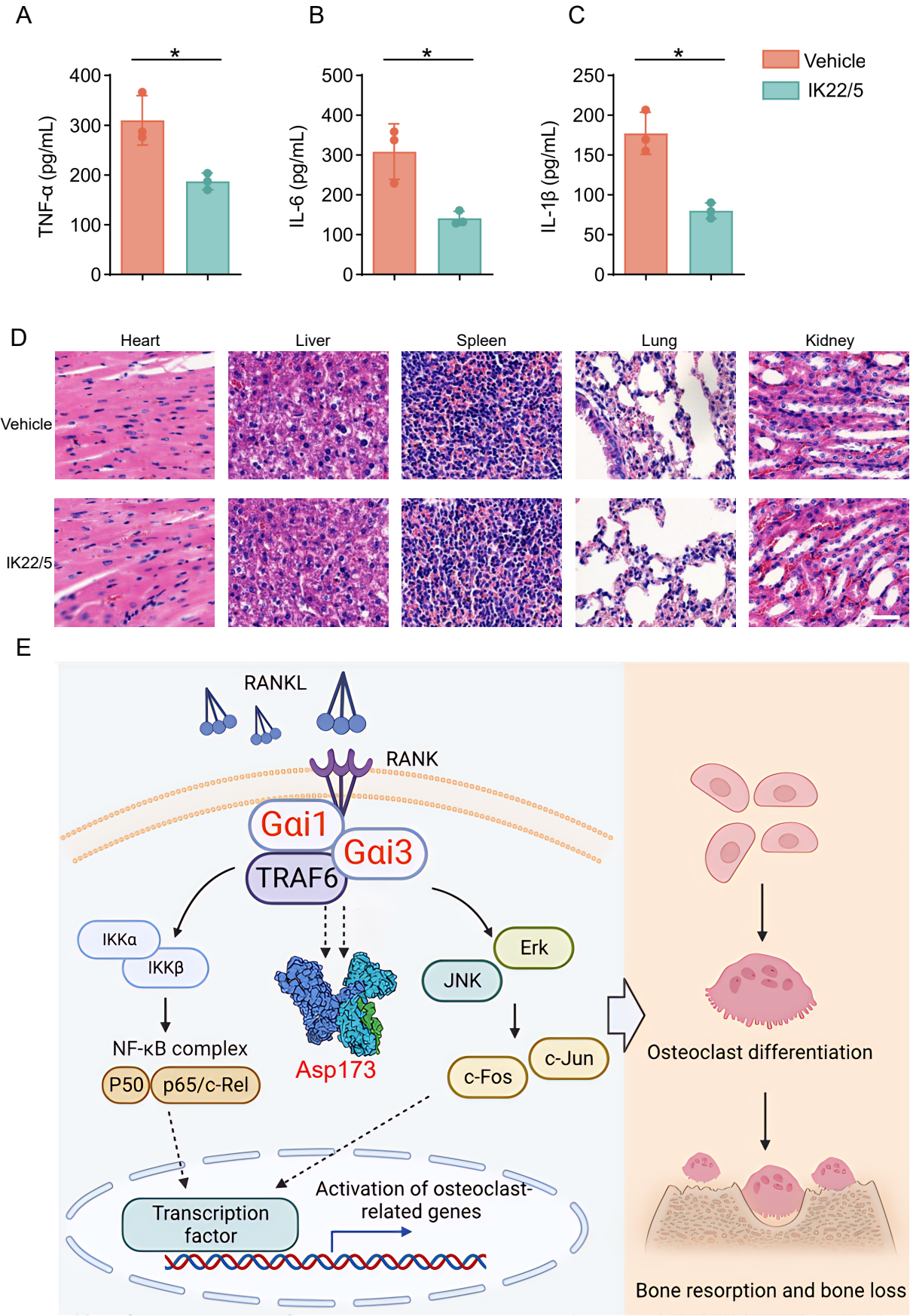

Supplement: Supplementary file 1 — Supporting File 1: advs74185‐sup‐0001‐FigureS1‐S7.pdf. [file ADVS-13-e10836-s004.pdf]
