# Supplementary material for: Gαi1/3 Is a Novel Regulatory Target for RANKL Signal Transduction and Osteoporosis
Source: Adv Sci (Weinh). 2026 Feb 12;13(20):e10836. doi: 10.1002/advs.202510836 (PMC13067840; doi:10.1002/advs.202510836)
Supplement: Supplementary file 2 — Supporting File 2: advs74185‐sup‐0002‐FigureCaptions.docx. [file ADVS-13-e10836-s001.docx]

**Fig. S1 Deletion of Gαi1/3 in myeloid cells alleviates bone loss induced by OVX.**

(A) Breeding strategy of *LysmCre; Gαi3^fl/fl^* mice. (B) Western blot analysis of Gαi1, Gαi2, and Gαi3 in *LysmCre; Gαi3^fl/fl^* BMMs. n = 3 per group*.* All bar graphs are presented as the mean ± SD. ns, p > 0.05; ***p < 0.001.(C-I) Representative μCT images and quantitative μCT analysis of cortical and trabecular bone microarchitecture in femora from mice in the *Gαi3^fl/fl^* and *LysmCre; Gαi3^fl/fl^* groups after sham and OVX operation (BMD, Bone Mineral Density; BV/TV, bone volume per tissue volume; Tb.N, trabecular number; Tb.Th, trabecular thickness; Tb.Sp, trabecular separation; Ct.Th, cortical bone thickness). n=5 per group.

(J-K) Representative images of TRAP staining in femora after sham and OVX operation and quantification of TRAP-positive cells in bone surface. Statistical analysis was performed using one-way ANOVA . n=5 per group. Scale bar: 50μm. NS, no significance, ****p* < 0.001.

**Fig. S2** **Gαi1/3 knockdown inhibits RANKL-induced osteogenesis**

(A-B) BMMs isolated from WT mice were infected with the lentiviral Gαi1 shRNA and the lentiviral Gαi3 shRNA. The cells were cultured in α-MEM medium supplemented with RANKL (50 ng/ml). On day 5, osteoclasts were stained with TRAP and quantification of osteoclast number. n=3 per group. Scale bar: 50μm.

(C-D) BMMs in different groups were cultured in α-MEM medium supplemented with RANKL(50 ng/ml). On day 5, osteoclasts were stained with phalloidin. Representative images of fluorescence of cell nuclei and F-actin, rings. green, actin ring; blue, DAPI. Quantification of percentage of osteoclast area. n=3 per group. Scale bar: 50μm. (E) Representative images of resorption pits on Osseo Assay surface. n=3 per group. Scale bar: 50μm.

(F) Osteoclasts were stained with NFATc1. Representative images of fluorescence of NFATc1. Green, FITC; red, NFATc1. n=3 per group. Scale bar: 10μm. (G) Quantification of resorption pit area. (H) Quantification of fluorescence intensity of NFATc1 in BMMs. (I-K) qPCR analysis of the osteoclast-specific genes mRNA expression of the BMM cultures in different groups incubated with RANKL (50 ng/ml) for 3 days. n=3 per group. Statistical analysis was performed using one-way ANOVA. Data were presented as mean ± SD. **p* < 0.05, ***P* < 0.01, ****p* <0.001.

**Fig. S3** **Gαi1/3 knockdown inhibits RANKL-induced downstream signal activation in BMMs.**

(A-F) BMMs in different groups were cultured, the proteins listed were examined by Western blotting and phosphorylation (vs total proteins) was quantified. n=3 per group. (G-J) Co-cultured BMMs with osteoblasts in a medium containing estrogen (E2, 1*10-7M, Sigma). Gαi1-3 of BMMs were examined by western blotting in different groups (Vehicle, 17β-estradiol ,Withdraw) . The expression level of Gαi1-3 was quantified. The expression level of Gαi1-3 was quantified. (K-L) Induce BMMs differentiate into osteoclasts. Osteoclasts in different groups were stained with TRAP and osteoclast number was quantified. n=3 per group. Scale bar: 50μm.

Statistical analysis was performed using one-way ANOVA. Data were presented as mean ± SD. NS, no significance, **p* < 0.05, ***p* < 0.01, ****p*<0.001.

**Fig. S4** **The impaired ability to form osteoclasts in Gαi1-shRNA and Gαi3-shRNA BMMs is rescued by the introduction of WT-Gαi1 or WT-Gαi3, respectively.**

(A-B) WT-Gαi1 or WT-Gαi3 were transfected into Gαi1-shRNA and Gαi3-shRNA BMMs, respectively.

TRAP staining was used to detect the osteoclast formation ability of each group, and osteoclasts nubmer and area of each group were quantified. n=3 per group. Scale bar: 100μm. (C) The expression of *Atp6v0d2, Ctsk* and *Mmp9* mRNA in cells of each group was detected by qPCR. Data were presented as mean ± SD. ****p* <0.001.

**Fig.** **S5** **RANKL-induced signaling and osteoclastic differentiation are inhibited in BMMs with Gαi1 and Gαi3 mutations.**

(A-B) BMMs with the dominant negative the Gαi3-173A construct (“dnGαi3”), or the vector control (“Vec”), were cultured in α-MEM medium supplemented with RANKL (50 ng/ml). On day 5, TRAP staining was used to detect the osteoclast formation ability of each group, and osteoclasts nubmer and area of each group were quantified. n=3 per group. Scale bar: 50μm. (C-D) Osteoclasts were stained with phalloidin. Representative images of fluorescence of cell nuclei and F-actin rings. green, actin ring; blue, DAPI. Osteoclast area was quantified for each group. n=3 per group. Scale bar: 50μm. (E) BMMs in different groups were cultured on Osteo Assay surface for 5 days. Representative images of resorption pits on Osseo Assay surface. n=3 per group. Scale bar: 50μm. (F) BMMs in different groups were treated with RANKL. On day 1, osteoclasts were stained with NFATc1. Representative images of fluorescence of NFATc1. Green, FITC; red, NFATc1. n=3 per group. Scale bar: 50μm. (G) Quantification of resorption pit area of “E”. (H) Quantification of fluorescence intensity of NFATc1 in BMMs of “F”. (I-K) qPCR analysis of mRNA expression of osteoclast-specific genes in BMM cultured from different groups after incubation with RANKL (50 ng/ml) for 3 days. n=3 per group.

(L-Q) BMMs in different groups were cultured in α-MEM medium supplemented with RANKL(50 ng/ml) for 25 or 50 min, the proteins listed were examined by Western blotting and phosphorylation was quantified. n=3 per group. Statistical analysis was performed using one-way ANOVA. Data were presented as mean ± SD. NS, no significance, ***p* < 0.01, ****p* <0.001.

**Fig.** **S6** **Transfection of WT-Gαi3 into Gαi3-mutant BMMs restores their capacity to differentiate into osteoclasts.**

(A-B) WT-Gαi3 was transfected into Gαi3-mutant BMMs, and then BMMs were induced to differentiate into osteoclasts. Representative images of TRAP staining in each group, and osteoclasts area of each group were quantified. n=3 per group. Scale bar: 100μm. (C) The expression of *Atp6v0d2, Ctsk* and *Mmp9* mRNA in cells of each group was detected by qPCR. (D) Osteoclast differentiation-related proteins (MMP9, CFOS, CTSK) were examined by Western blotting. Statistical analysis was performed using one-way ANOVA. Data were presented as mean ± SD. **p* <0.05, ***p* <0.01, ****p* <0.001.

**Fig.** **S7** Administration of IKK22/5 did not produce obvious side effects in mice，and the pattern of Gαi1/3 regulation in RANKL signal transduction and osteoclastogenesis.(A-C) Serum levels of TNF-α, IL-6, and IL-1β in OVX mice of different groups. (D) Representative images of H&E staining of heart, liver, spleen, lung and kidney. (E) The pattern of Gαi1/3 regulation in RANKL signal transduction and osteoclastogenesis. The Gαi1/3 protein is a key linker protein for RANKL signal transduction. It activates downstream signals such as NF-κB and MAPK by mediating the binding of RANK and TRAF6. This further plays a role in promoting the differentiation of osteoclasts. Among them, the 173 Asp residue of Gαi1/3 was a critical residue for mediating RANK-TRAF6 binding. n=5 per group. Scale bar: 200μm. Statistical analysis was performed using one-way ANOVA. Data were presented as mean ± SD. **p* <0.05.
