# Supplementary material for: Gαi1/3 Is a Novel Regulatory Target for RANKL Signal Transduction and Osteoporosis
Source: Adv Sci (Weinh). 2026 Feb 12;13(20):e10836. doi: 10.1002/advs.202510836 (PMC13067840; doi:10.1002/advs.202510836)
Supplement: Supplementary file 3 — Supporting File 3: advs74185‐sup‐0003‐Antibodies.docx. [file ADVS-13-e10836-s003.docx]

| Number | Antibodies | SOURCE | IDENTIFIER |
| --- | --- | --- | --- |
| 1 | Gαi1 | Santa Cruz Biotech | sc-13533 |
| 2 | Gαi2 | Santa Cruz Biotech | sc-13534 |
| 3 | Gαi3 | Santa Cruz Biotech | sc-365422 |
|  | HRP IgG | Santa Cruz Biotech | sc-2030/2031 |
| 4 | GAPDH | Cell Signaling Tech | #5174 |
| 5 | β-Tubulin | Cell Signaling Tech | #15115 |
| 6 | JNK | Cell Signaling Tech | #9252 |
| 7 | p-JNK | Cell Signaling Tech | #4668 |
| 8 | Erk1/2 | Cell Signaling Tech | #9102 |
| 9 | p-Erk1/2 | Cell Signaling Tech | #9101 |
| 10 | p38 | Cell Signaling Tech | #9212 |
| 11 | p-p38 | Cell Signaling Tech | #4511 |
| 12 | Akt123 | Cell Signaling Tech | #2967 |
| 13 | pAkt-S473 | Cell Signaling Tech | #9271 |
| 14 | p65 | Cell Signaling Tech | #8242 |
| 15 | p-p65 | Cell Signaling Tech | #3033 |
|  | NFATc1 | Cell Signaling Tech | #5861 |
| 16 | RANK | Abcam | Ab13918 |
| 17 | TRAF6 | Abcam | Ab40675 |
| 18 | Grb2 | Abcam | Ab32037 |
| 19 | MMP9 | Abcam | Ab76003 |
| 20 | cFOS | Abcam | Ab302667 |
| 21 | Ctsk | Abcam | Ab187647 |

**Information of Antibodies**
