# Supplementary material for: Gαi1/3 Is a Novel Regulatory Target for RANKL Signal Transduction and Osteoporosis
Source: Adv Sci (Weinh). 2026 Feb 12;13(20):e10836. doi: 10.1002/advs.202510836 (PMC13067840; doi:10.1002/advs.202510836)

FIG 1

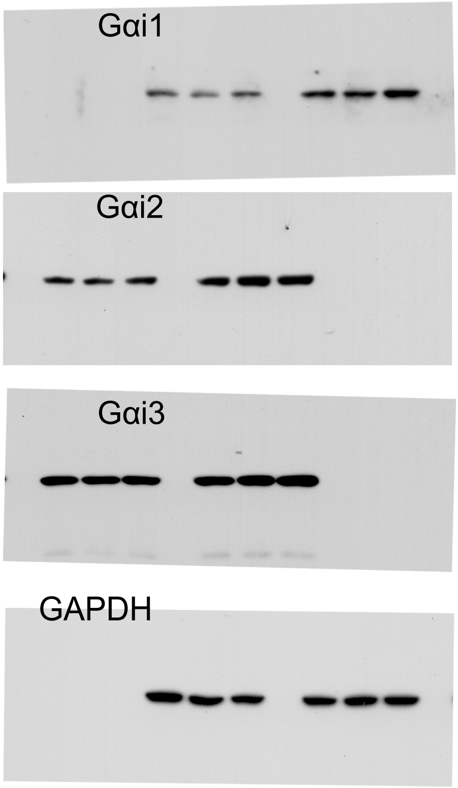

FIG 2

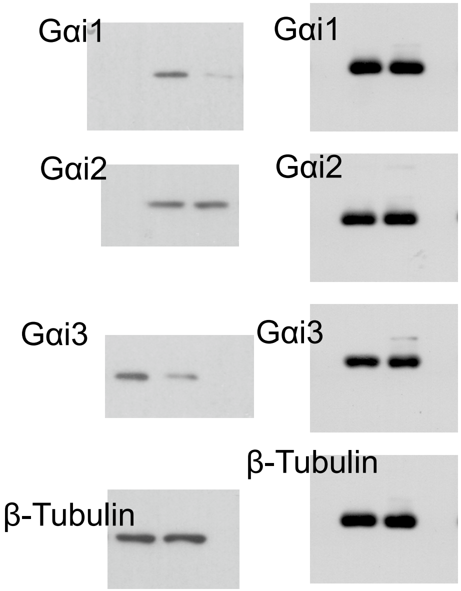

FIG S1

FIG S1

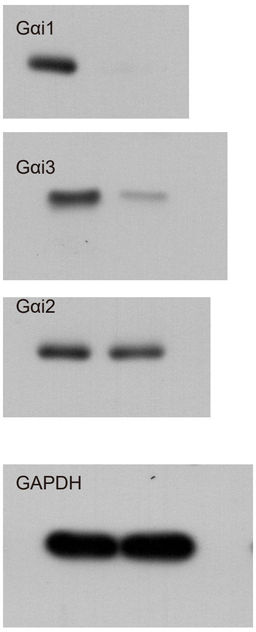

FIG 4

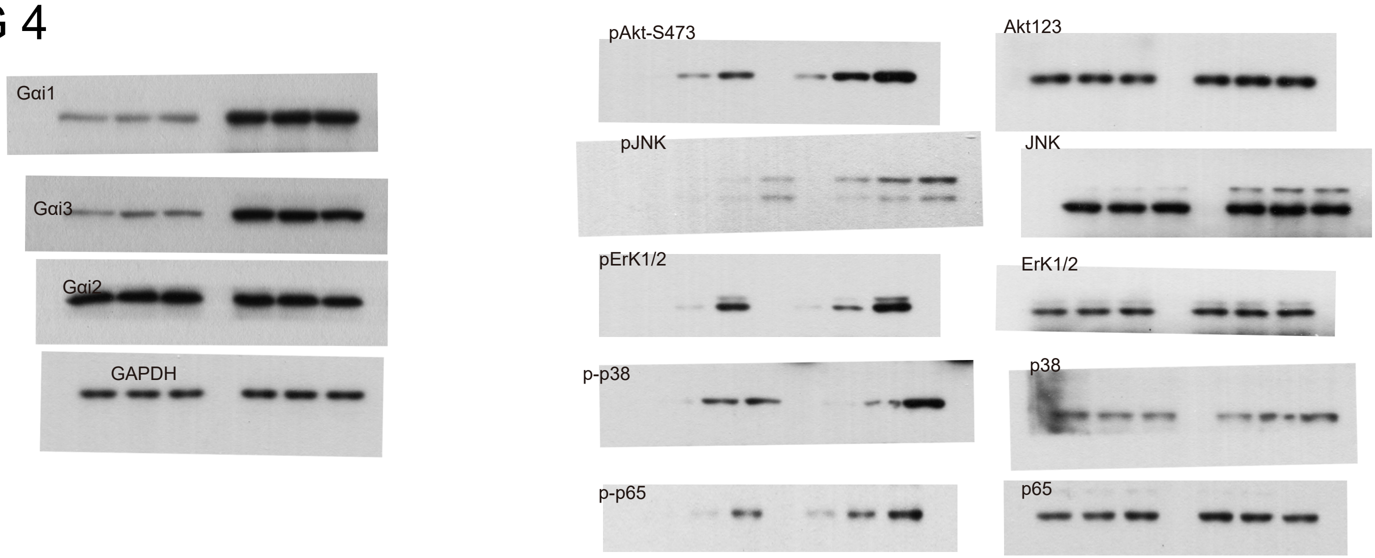

FIG S3

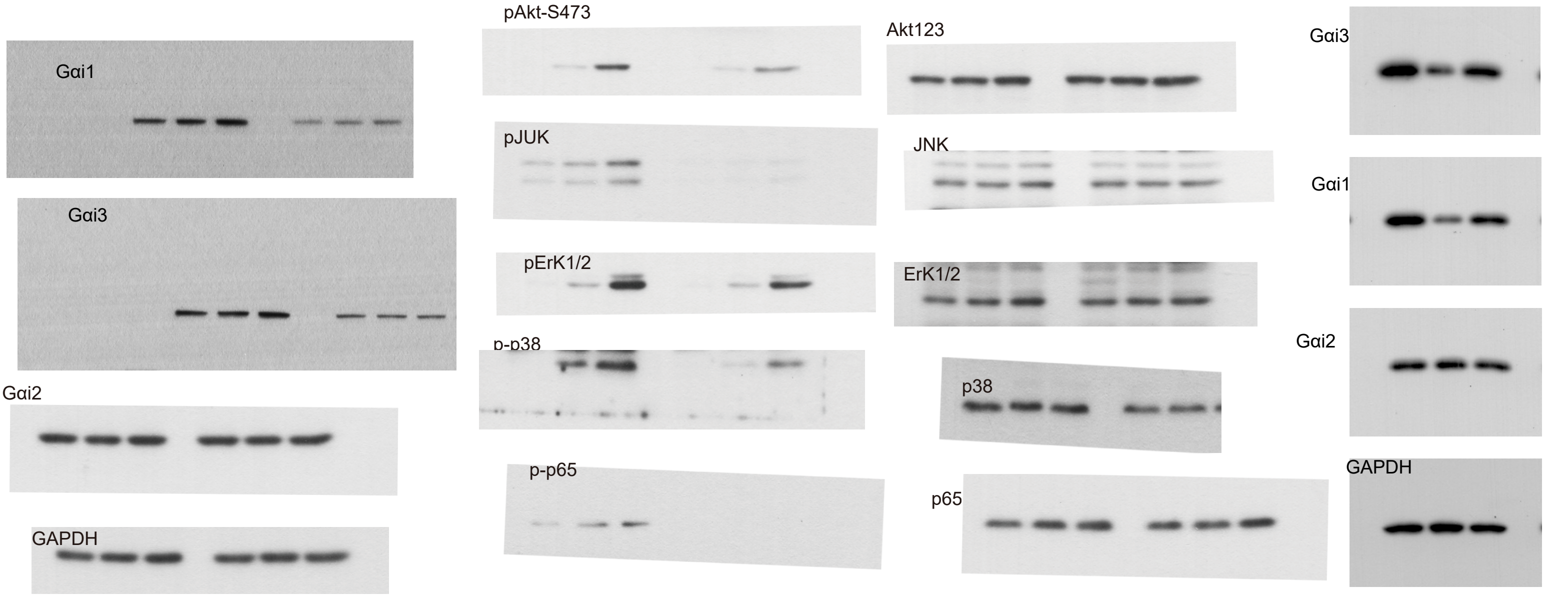

FIG 5 A-C

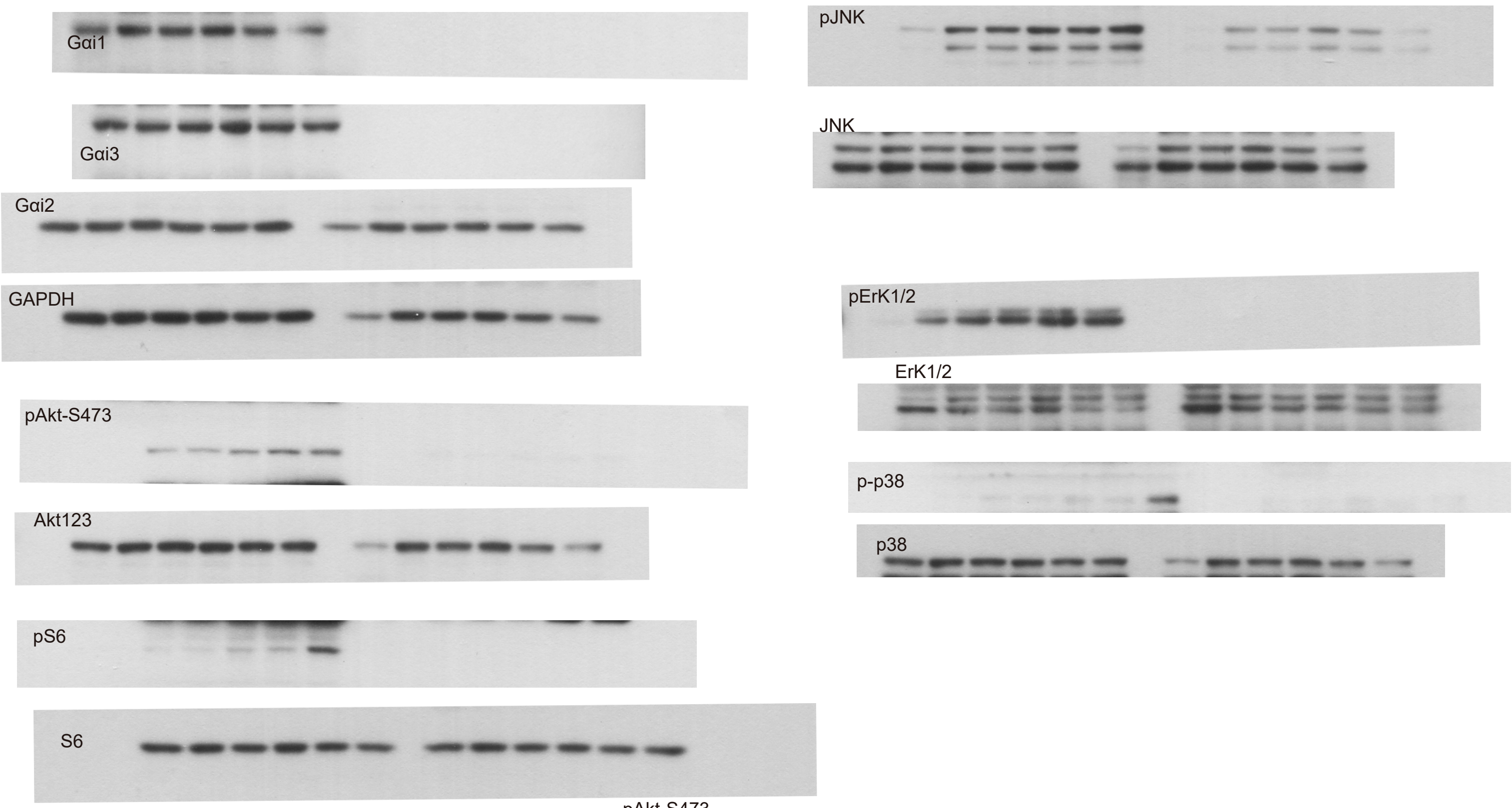

FIG 5 F-H

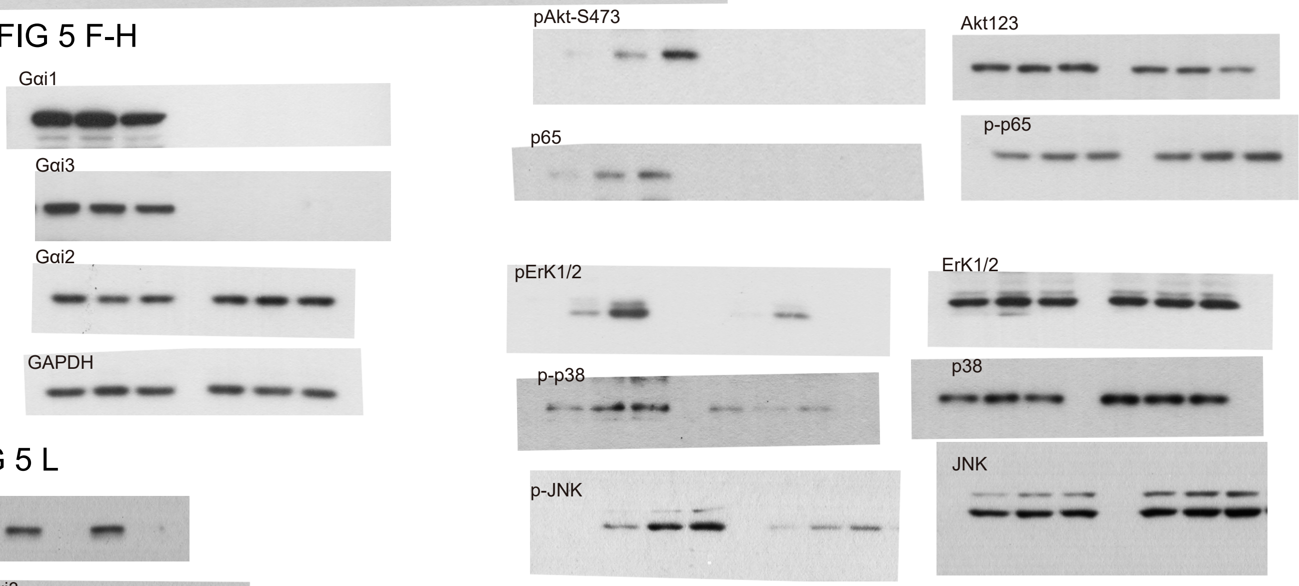

FIG 5 L

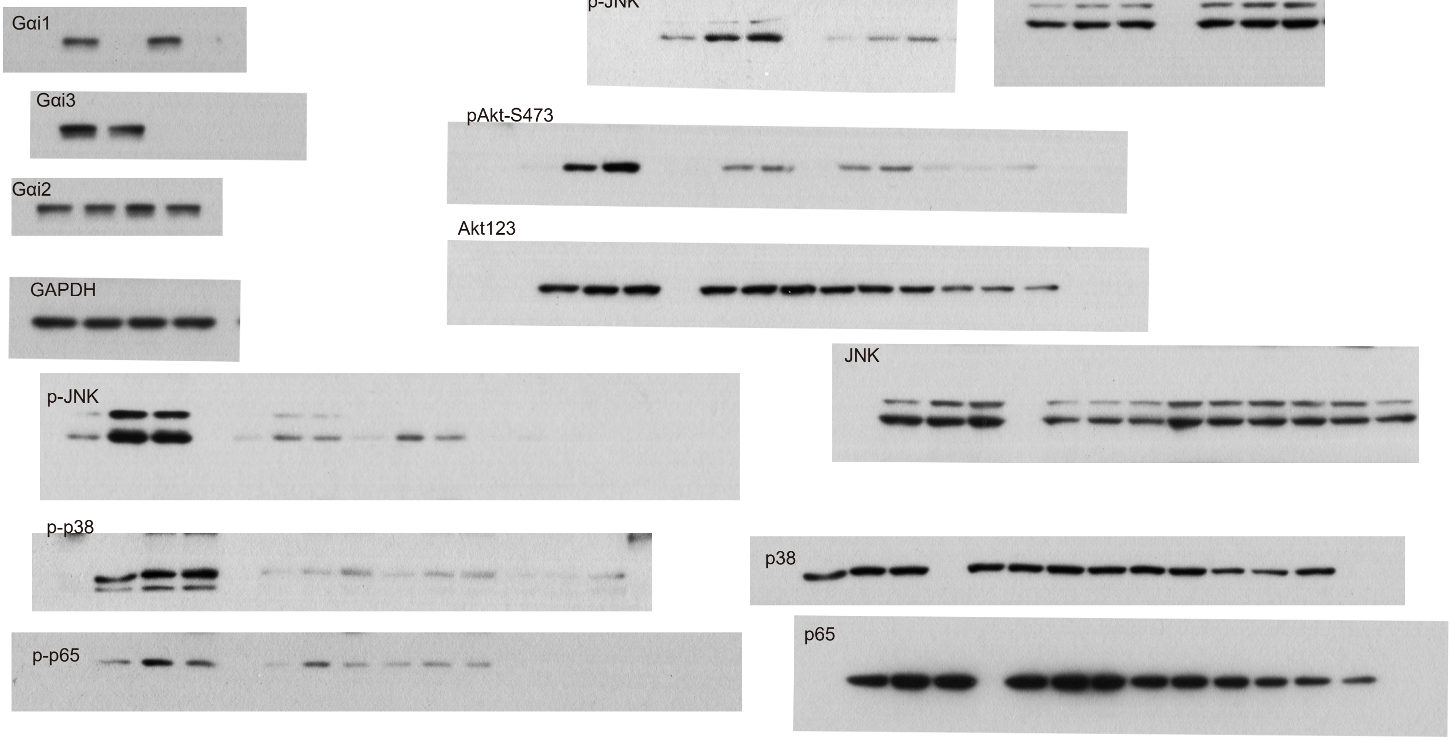

FIG 6A

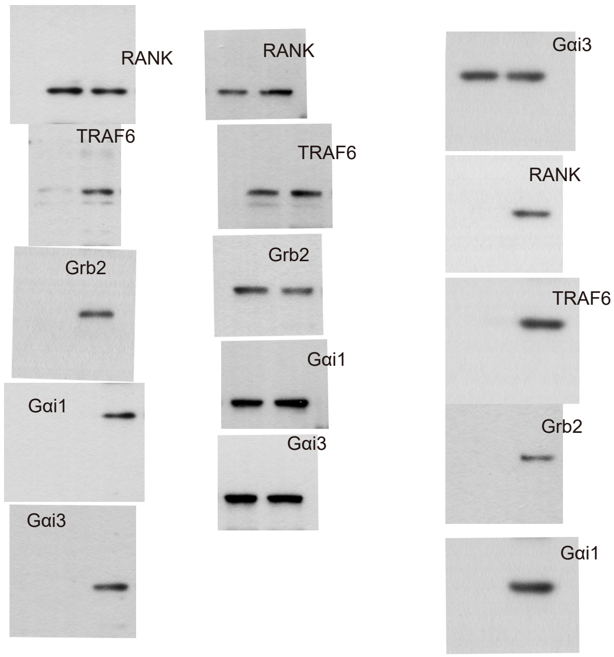

FIG 6E

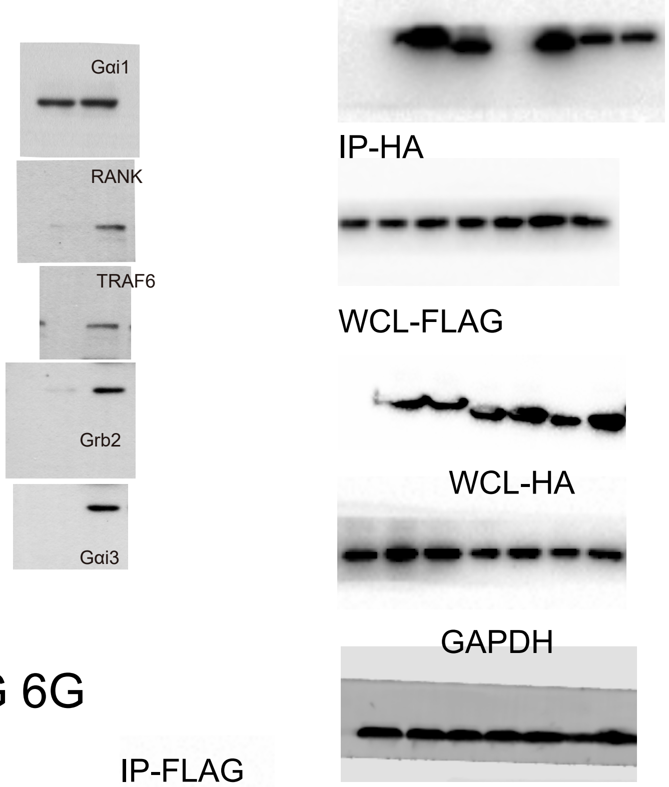

FIG 6F

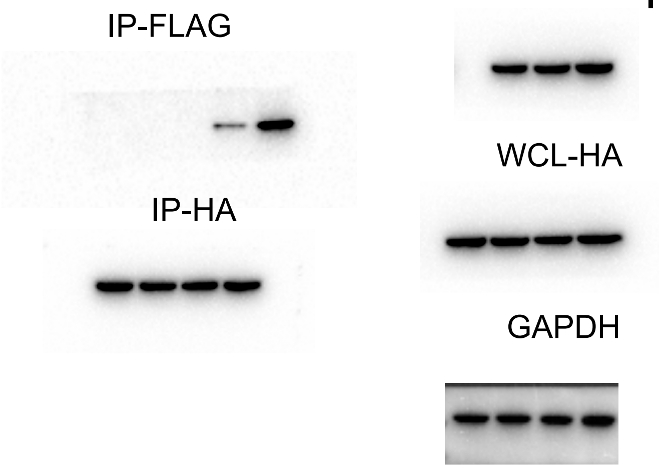

FIG 6G

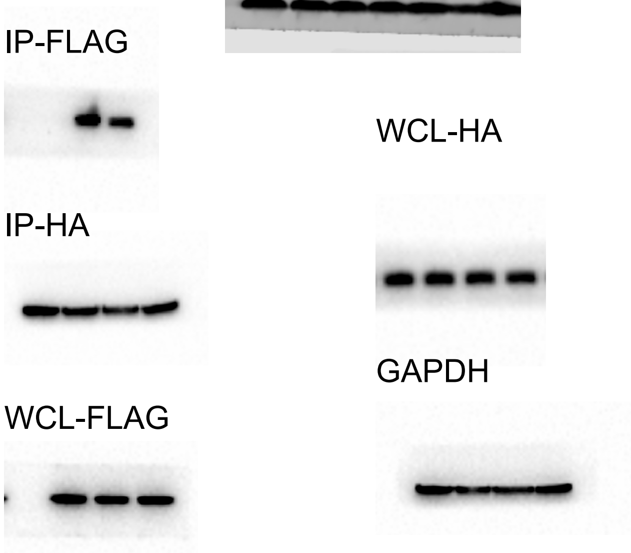

FIG S5

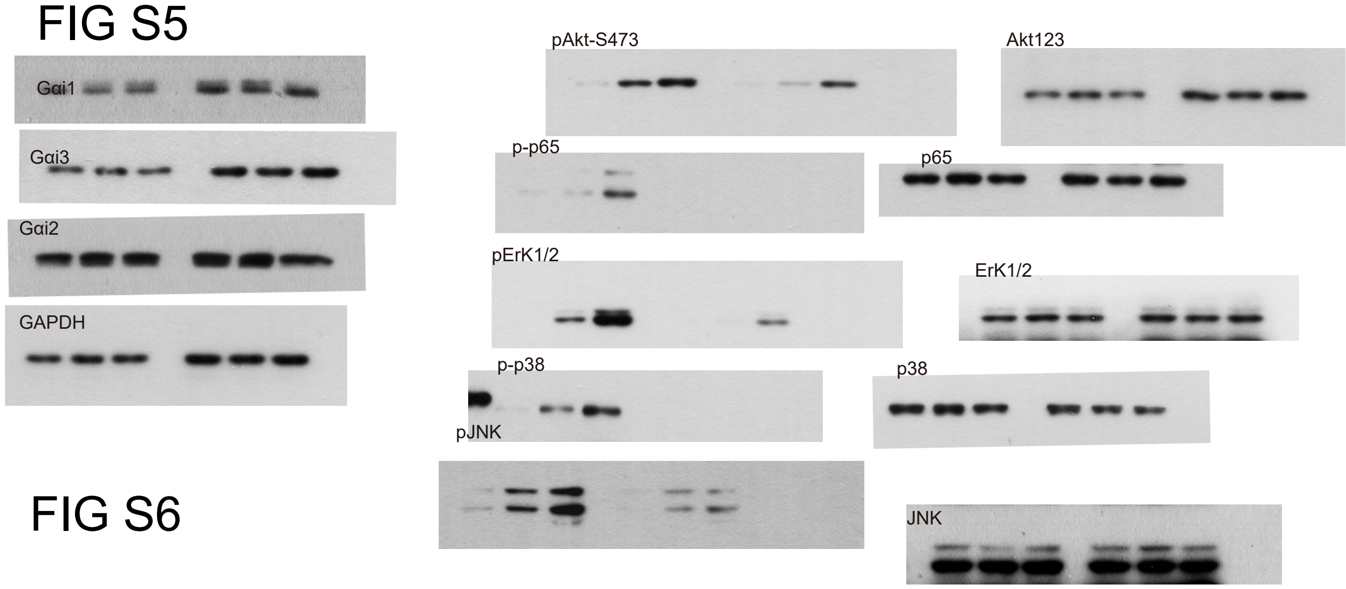

FIG S6

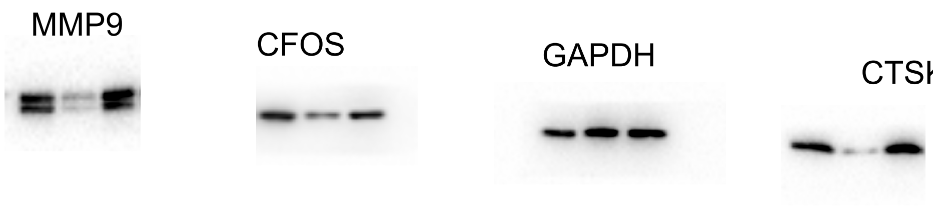

Supplement: Supplementary file 4 — Supporting File 4: advs74185‐sup‐0004‐ImageofBlots.pdf. [file ADVS-13-e10836-s002.pdf]
